# A drug-repurposing nanotheranostic for synergistic management of resistant bacterial pneumonia

**DOI:** 10.1016/j.ajps.2026.101185

**Published:** 2026-07-11

**Authors:** Xianbin Sun, Xinyi Wang, Fangying Zheng, Ruofei Xu, Xinyi Shen, Ding Tan, Xudong Li, Ya Wang, Kaiming Peng, Haijun Chen, Xiumei Li, Yu Gao

**Affiliations:** a Fujian Provincial Key Laboratory of Cancer Metastasis Chemoprevention and Chemotherapy, College of Chemistry, Fuzhou University, Fuzhou, 350108, China; b Department of Radiology, The First Affiliated Hospital of Fujian Medical University, Fuzhou, 350005, China; c Department of Thoracic Surgery, Fujian Medical University Union Hospital, Fuzhou, 350001, China

**Keywords:** Bacterial pneumonia, Inflammatory microenvironment, Theranostic nanoplatform, Tetracycline, Baicalin, Dextran

## Abstract

The rising threat of antibiotic resistance severely compromises the treatment of bacterial pneumonia, underscoring the urgent need for innovative and comprehensive therapeutic strategies. Here, we developed an innovative theranostic nanoplatform (Dex/BTGd) through the coordinated assembly of tetracycline, gadolinium ion (Gd^3+^) and baicalin, which acts synergistically to combat infection, enable non-invasive magnetic resonance imaging (MRI), and regulate the lung microenvironment. Dex/BTGd demonstrated potent antibacterial activity against clinically relevant pathogens, effectively disrupting biofilms and overcoming tetracycline resistance. The incorporated Gd^3+^ allowed targeted visualization of pulmonary infection sites by MRI, facilitating timely disease assessment. Beyond its direct antibacterial effects, the nanoplatform modulated the inflammatory microenvironment by scavenging reactive oxygen species, promoting macrophage polarization from the pro-inflammatory to the anti-inflammatory phenotype, and suppressing profibrotic transforming growth factor-β signaling. In a murine model of tetracycline-resistant *Pseudomonas aeruginosa* pneumonia, Dex/BTGd significantly reduced bacterial load, alleviated pulmonary edema and fibrosis, and enabled targeted MRI visualization of infected lesions. This integrated strategy of pathogen clearance, microenvironment modulation, and tissue repair offers a multifaceted solution to drug-resistant bacterial pneumonia, providing a promising blueprint for next-generation anti-infective systems.

## Graphical abstract

**Figure:**
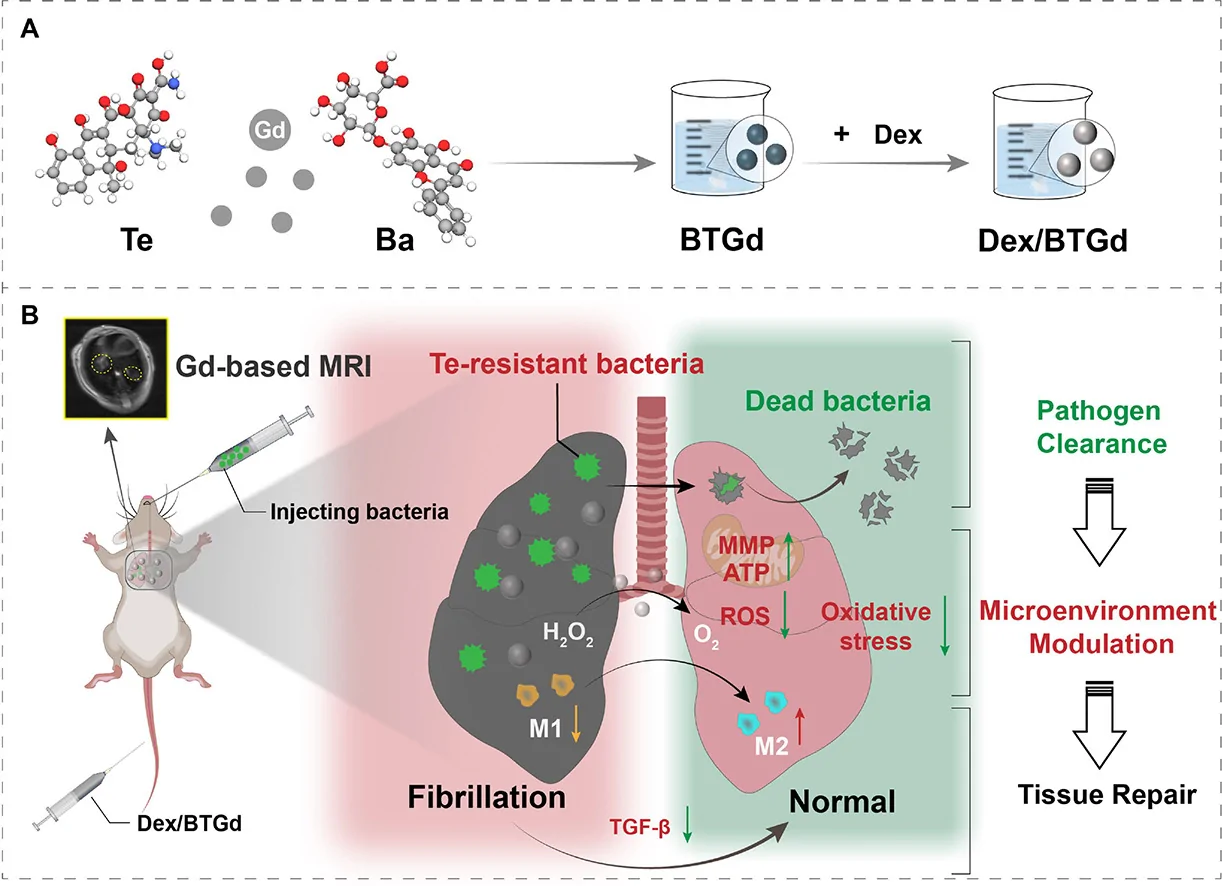

## 1 Introduction

Lower respiratory tract infections rank among the deadliest infectious diseases worldwide [1], and bacterial pneumonia represents the most severe clinical form [2]. Although antibiotics remain the primary clinical treatment, their overuse has led to a sharp rise in bacterial resistance, posing a grave global health threat. It is projected that drug-resistant infections may cause up to 39 million deaths worldwide between 2024 and 2050, accompanied by enormous economic losses [3]. The emergence of "superbugs'' and biofilm formation, which can increase antibiotic tolerance by 100 to 1000 times, further complicates treatment [4], underscoring the urgent need for innovative anti-infective strategies.

Bacterial pneumonia often triggers inflammatory responses characterized by chemokine release and immune cell infiltration. While immune cell infiltration is essential for pathogen clearance [5], overactivation can result in cytokine storms and irreversible histopathological damage. Abnormal immune activation and the resulting cytokine storm are associated with increased mortality risk [6]. Persistent pro-inflammatory (M1) macrophage activation and excessive secretion of transforming growth factor-β (TGF-β) contribute to pulmonary fibrosis [7]. Additionally, bacterial and immune-derived reactive oxygen species (ROS) exacerbate oxidative stress [8], creating a microenvironment that not only damages host tissues but also imposes selective pressure on bacteria. Growing evidence indicates that sublethal ROS levels drive bacterial adaptation and antibiotic resistance by inducing stress repair pathways, upregulating efflux pump expression, and promoting biofilm formation [9]. Meanwhile, the inflammatory microenvironment further reinforces these processes, creating a feedback loop that enhances bacterial survival and persistence [10]. Recent studies highlight the therapeutic benefits of combining antibacterial and anti-inflammatory approaches. For instance, Chen et al*.* used oxygen-deficient WO_x_ nanobelts to achieve synergistic antibacterial and anti-inflammatory effects in bone infection models through photothermal sterilization and by promoting macrophage polarization [11]. Similarly, Ye et al. designed a ROS-scavenging nanofiber dressing that effectively suppressed excessive inflammatory responses and accelerated healing in diabetic wounds [12].

Accurate diagnosis is equally critical in managing pulmonary infections. However, conventional methods, such as traditional etiological diagnostic methods and molecular biology techniques, suffer from either low sensitivity [13] or operational complexity [14]. Medical imaging technology plays an irreplaceable role in early diagnosis and disease assessment. Computed tomography (CT) is currently the "gold standard'' for thoracic imaging, offering high resolution but carrying the risk of ionizing radiation, which has been linked to increased cancer risk [15]. In contrast, magnetic resonance imaging (MRI) provides superior soft-tissue contrast without radiation exposure. Although gadolinium (Gd)-based contrast agents play a crucial role in enhancing MRI resolution in clinical practice, they carry risks of nephrogenic fibrosis [16] and lack therapeutic function, driving the need for safer and multifunctional alternatives.

**Scheme 1:**
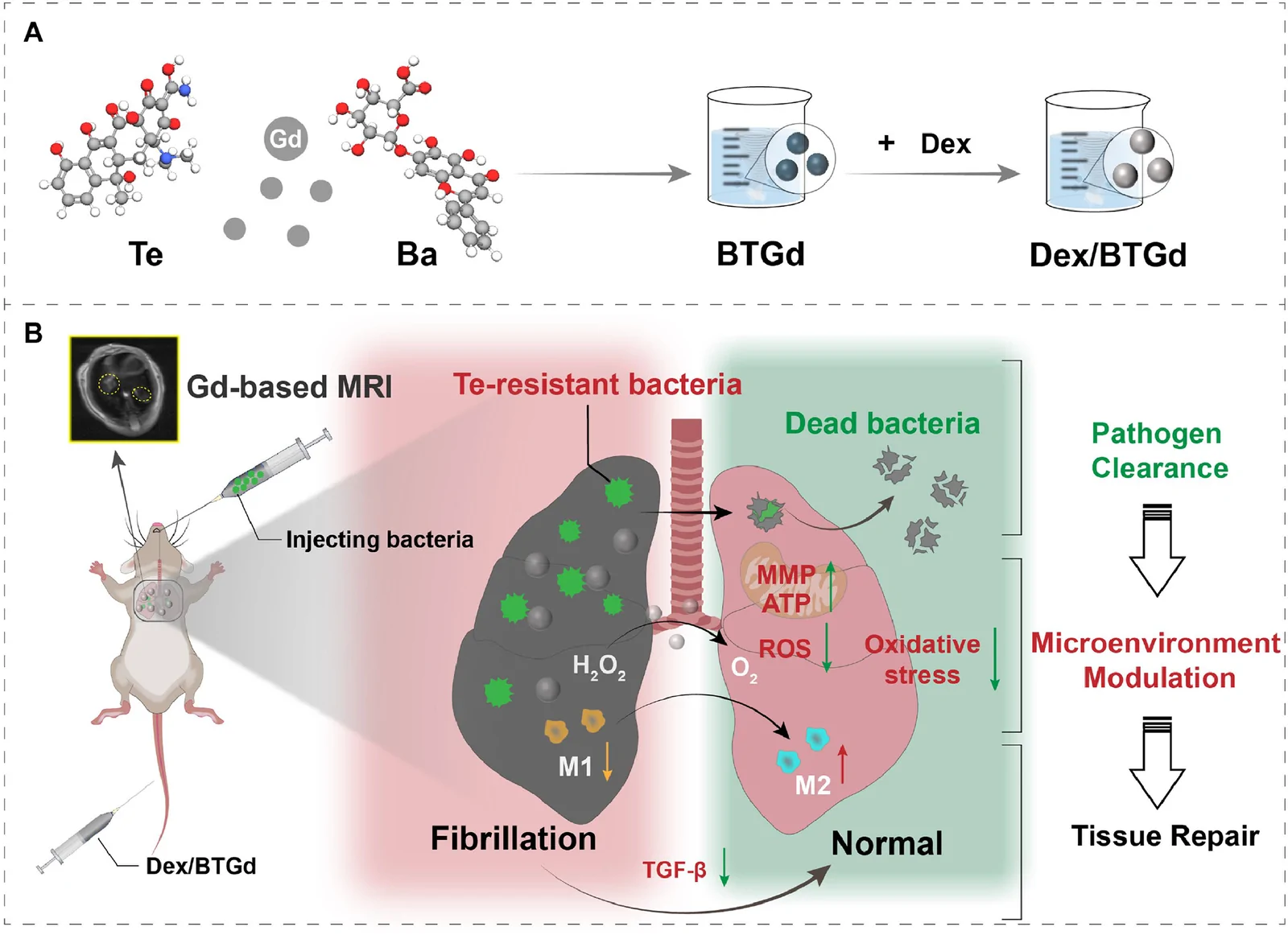
The schematic illustration of the preparation (A) and therapeutic mechanisms (B) of Dex/BTGd nanoplatform.

To address these challenges, we constructed a theranostic nanoplatform, Dex/BTGd, via coordination-driven self-assembly of baicalin (Ba), tetracycline (Te) and Gd^3+^, followed by surface modification with dextran (Dex) to improve stability and biofilm targeting [17]. Specifically, Ba is a bioactive flavonoid from *Radix scutellariae* that exhibits intrinsic antibacterial, antioxidant and anti-inflammatory properties. Its carbonyl and phenolic hydroxyl groups enable metal coordination, with previous studies showing significantly enhanced efficacy upon metal complexation [18]. Te, although facing widespread resistance [19], retains metal-chelating ability that offers potential for resistance reversal [20]. Notably, our previous study demonstrated its potent anti-melanoma activity through copper ion chelation [21]. Meanwhile, Gd^3+^ enables high-resolution MRI. In this system, the three components work in concert to exert synergistic antibacterial, antioxidant and inflammation-modulating effects (Scheme 1). *In vitro* studies demonstrated that Dex/BTGd can effectively eradicate bacteria, suppress biofilm formation, mitigate oxidative stress, and promote M2 macrophage polarization. In a murine model of drug‑resistant bacterial pneumonia, Dex/BTGd achieved favorable therapeutic outcomes by integrating lesion diagnosis with multifaceted anti-infection strategies into a coordinated pathogen clearance, microenvironment modulation, and tissue repair approach.

## 2 Materials and methods

### 2.1 Materials

Te, Dex, 3,3′,5,5′-tetramethylbenzidine (TMB), 2,2′-azino-bis (3-ethylbenzothiazoline-6-sulfonic acid) (ABTS) and mangiferin were obtained from Energy Chemical Technology Co., Ltd. (Shanghai, China). Ba, 1,1-diphenyl-2-picrylhydrazyl (DPPH), urea and Tween 20 were obtained from Macklin Biochemical Technology Co., Ltd. (Shanghai, China). Sodium dodecyl sulfate (SDS) and fetal bovine serum (FBS) were purchased from Tagene Biotechnology Co., Ltd. (Xiamen, China). Gadolinium (III) chloride hexahydrate (GdCl_3_·6H_2_O), ethylenediaminetetraacetic acid (EDTA), doxycycline and oxytetracycline were obtained from Aladdin Biochemistry Technology Co., Ltd. (Shanghai, China). Potassium persulfate (K_2_S_2_O_8_), sulfanilic acid (C_6_H_7_NO_3_S), *N*-1-naphthylethylenediamine dihydrochloride (C_12_H_16_Cl_2_N_2_), sodium nitrite (NaNO_2_), ammonium molybdate tetrahydrate [(NH_4_)_6_Mo_7_O_24_·4H_2_O], hydrogen peroxide (H_2_O_2_) and liquid paraffin were obtained from Sinopharm Group Co., Ltd. (Shanghai, China). Gadoteric acid meglumine salt injection (Gd-DOTA) was obtained from Hengrui Pharmaceuticals Co., Ltd. (Jiangsu, China). Epigallocatechin gallate was purchased from Meryer Chemical Technology Co., Ltd. (Shanghai, China). Quercetin was bought from Titan Scientific Co., Ltd. (Shanghai, China). Luria-Bertani (LB) broth and LB agar powder were purchased from Huangkai Bionotechnology Co., Ltd. (Guangdong, China). Crystal violet staining solution, 2′,7′-dichlorodihydrofluorescein diacetate (DCFH-DA), Rhodamine 123, Annexin V-fluorescein isothiocyanate (FITC)/propidium iodide (PI) apoptosis detection kit and LIVE/DEAD bacterial staining kit with dimethylaminooxonol (DMAO) & PI were obtained from Beyotime Biotechnology Co., Ltd. (Shanghai, China). Penicillin and streptomycin were obtained from New Cell & Molecular Biotech Co., Ltd. (Suzhou, China). Tris (4,7-diphenyl-1,10-phenanthroline) ruthenium (II) dichloride ([Ru(dpp)_3_]Cl_2_) was purchased from Maokang Biotechnology Co., Ltd. (Shanghai, China). Hoechst 33342 was obtained from Thermo Fisher Scientific (USA). Tannic acid, 3-(4,5-dimethylthiazol-2-yl)-2,5-diphenyltetrazolium bromide (MTT), lipopolysaccharide (LPS) and interferon-γ (IFN-γ) were purchased from Meilun Biotechnology Co., Ltd. (Dalian, China). Interleukin-4 (IL-4) was obtained from Novoprotein Scientific Co., Ltd. (Suzhou, China). Anti-mouse CD80-allophycocyanin (APC), anti-mouse CD206-phycoerythrin (PE) and anti-mouse CD11b-FITC were purchased from BioLegend Company (USA). ATP assay kit was obtained from Abbkine Scientific Co., Ltd. (Wuhan, China). Sodium pentobarbital was bought from Dingguochangsheng Biotechnology Co., Ltd. (Beijing, China). Hematoxylin and Eosin (H&E) staining solution was purchased from Biosharp Life Science (Hefei, China). Masson's trichrome staining kit and malondialdehyde (MDA) content assay kit were obtained from Solarbio Life Science Co., Ltd. (Beijing, China). Mouse TGF-β enzyme-linked immunosorbent assay (ELISA) kit was purchased from MultiSciences (Hangzhou, China). Glutamic pyruvic transaminase (ALT/GPT) and glutamic oxaloacetic transaminase (AST/GOT) assay kits were purchased from Nanjing Jiancheng Bioengineering Research Institute (Nanjing, China). Creatinine (CRE) and urea nitrogen assay kits were obtained from Boxbio Co., Ltd. (Beijing, China). Dexamethasone sodium phosphate injection was purchased from Cisen Pharmaceutical Co., Ltd. (Jining, China). NIH/3T3 fibroblast and RAW 264.7 monocyte cell lines were purchased from the Cell Resource Center of Shanghai Institute for Biological Sciences (Chinese Academy of Sciences).

### 2.2 Self-assembly and characterization

Te, Ba, Te–Ba (a mixture of Te and Ba), Ba–Gd (a mixture of Ba and Gd^3+^), Te–Gd (a mixture of Te and Gd^3+^), and Ba–Gd–Te (a mixture of Te, Ba and Gd^3+^, named as BTGd) were prepared. The concentrations of Te and Ba were 1 mg/mL, and the concentration of Gd^3+^ was 1 mM. The mixtures were stored at 4 °C for 4 h. Particle size was measured by dynamic light scattering (DLS) using a Zetasizer Nano ZS instrument (Malvern, UK). Additionally, the volume of Gd^3+^ was varied (200, 150, 100, 50 and 25 µl) while keeping other components constant. The ultraviolet-visible (UV–Vis) absorption spectra of each sample were recorded on a Shimadzu UV-2700 spectrophotometer (Japan).

### 2.3 Investigation of self-assembly driving forces

BTGd was added into aqueous systems containing different concentrations of DMSO (5%, 10%, 15%, 20%, v/v), SDS (0.2%, 0.1%, 0.05%, 0.025%, w/v), EDTA (10, 20, 50, 100 mM), NaCl (100, 200, 500, 1000 mM), Tween 20 (10, 20, 50, 100 mM), or urea (10, 20, 50, 100 mM). Each concentration series was labeled as Treat 1 to 4. The changes in particle size were measured using DLS.

### 2.4 Preparation and characterization of Dex/BTGd

Ba (1 mg/ml, 1 ml), Te (1 mg/ml, 1 ml) and Gd^3+^ (1 mM, 1 ml) were combined and incubated at 4 °C for 4 h. Subsequently, 1 mL Dex (1 mg/ml) was added, and the mixture was further incubated overnight at 4 °C. The resulting solution was then transferred into a dialysis bag (MWCO 7 kDa) and dialyzed for 6 h to remove residual DMSO, affording Dex/BTGd. If necessary, the sample was concentrated using a 3 kDa ultrafiltration tube (1000 rpm, 5 min).

The hydrodynamic diameter and zeta potential of BTGd and Dex/BTGd were determined by DLS. The morphology of the nanocomplexes was characterized by transmission electron microscopy (TEM, Hitachi, Japan). The variation in particle size of Dex/BTGd and BTGd dispersed in phosphate-buffered saline (PBS) at 4 °C was monitored over 3 days to evaluate their colloidal stability. The UV–Vis absorption spectra of BTGd and Dex/BTGd were recorded using a UV–Vis spectrophotometer.

To evaluate the general applicability of the assembly strategy, the same preparation procedure described above was used. Mangiferin, quercetin, epigallocatechin gallate (EGCG), and tannic acid were used to replace Ba in the nanocomplexes at identical concentrations and volumes, yielding formulations denoted as T1, T2, T3 and T5, respectively, while the original Dex/BTGd formulation was designated as T4. All other preparation parameters were kept constant, and the hydrodynamic diameter and polydispersity index (PDI) of each formulation were measured by DLS. In addition, oxytetracycline (O1, O2, O3, O4, O5) or doxycycline (D1, D2, D3, D4, D5) at 1 mg/ml was used to replace the Te component in the T1-T5 formulations at an equivalent dose, respectively, with all remaining preparation conditions held unchanged.

### 2.5 Gd^3+^ release

Standard solutions were prepared by dissolving GdCl_3_·6H_2_O in 1% (v/v) nitric acid to achieve Gd^3+^ concentrations of 0.5, 1.0, 2.0, 4.0, 8.0 and 10.0 µg/ml. Aliquots (5 mL) of GdCl_3_·6H_2_O, Dex/BTGd and BTGd (each diluted in PBS and adjusted to contain 62.9 µg/ml Gd^3+^) were transferred into separate dialysis bags (MWCO 3 kDa). The bags were sealed, placed in 50 mL centrifuge tubes containing 20 mL deionized water, and incubated in a thermostatic shaker at 37 °C and 120 rpm. After 24 h, the external dialysate was collected and acidified with 1% nitric acid, and the released Gd^3+^ concentration was quantified by inductively coupled plasma optical emission spectrometry (ICP–OES, Thermo Fisher iCAP 7400, USA). Simultaneously, the hydrodynamic diameter of Dex/BTGd and BTGd nanocomplexes retained in the dialysis bags was monitored by DLS. UV–Vis absorption spectra of the samples were recorded from 250 to 500 nm using a spectrophotometer.

### 2.6 In vitro MRI experiments

Dex/BTGd and Gd-DOTA were dispersed in physiological saline at Gd^3+^ concentrations of 0.20, 0.15, 0.10, 0.05, 0.025, 0.0125 and 0 µM. The dispersions were transferred to 1.5 mL centrifuge tubes and positioned in a sample holder for MRI acquisition. *T_1_*‑weighted images were obtained on a 3.0 T clinical MRI system (Siemens, Germany) using the following parameters: repetition time (TR) = 650 ms, echo time (TE) = 10 ms, slice thickness = 1.0 mm, slice gap = 1.0 mm, and field of view (FoV) = 80 × 80 mm^2^.

Signal intensities of Dex/BTGd and Gd‑DOTA at each Gd^3+^ concentration were quantified to calculate the longitudinal relaxivity (*r_1_*) and evaluate their *T_1_* contrast enhancement efficiency.

To assess the robustness of the imaging performance, the effects of different metal ions (5 mM K^+^, 145 mM Na^+^, 2.5 mM Ca^2+^ and 1.25 mM Mg^2+^) and pH conditions (pH = 7.0, 5.0, 7.3 and 8.5) on the relaxivity and MRI signal of Dex/BTGd were also investigated.

### 2.7 Free radical scavenging assays

#### 2.7.1 Hydroxyl radical (•OH) scavenging assay

A solution of FeSO_4_ (1 mM) and H_2_O_2_ (2 mM) in sodium acetate buffer was prepared at a 1:1 vol ratio. Equal volumes of Te, Ba, Dex/BTGd and ascorbic acid dispersions (100 µg/ml) were then added to this mixture. After incubation at 37 °C for 5 min, TMB (1 mM) was introduced and the reaction mixtures were allowed to stand for an additional 2 min. UV–Vis absorption spectra in the range of 550 to 800 nm were subsequently recorded using a spectrophotometer.

#### 2.7.2 DPPH radical (DPPH•) scavenging assay

Te, Ba, Dex/BTGd and ascorbic acid dispersions (100 µg/ml, 1.0 ml) were each mixed with an equal volume of DPPH solution (0.10 mM). The mixtures were incubated in the dark at 37 °C for 30 min, after which the absorbance was recorded at 517 nm. The DPPH• scavenging activity (%) was calculated using the following equation:

(1) $$DPPH•\;scavenging\;rate\;\left(\%\right)=\left[1-\frac{A_{1}-A_{2}}{A_{0}}\right]\times \;100\%$$

where *A_1_, A_2_* and *A_0_* are the absorbance of the sample, the sample blank, and the control, respectively.

#### 2.7.3 ABTS radical (ABTS•^+^) scavenging assay

ABTS solution (7 mM) was mixed with an equal volume of K_2_S_2_O_8_ (4.9 mM) and allowed to stand overnight at room temperature in the dark to generate the ABTS•^+^ working solution. The absorbance was then adjusted to 0.70 ± 0.02 at 734 nm using PBS. Subsequently, equal volumes of the ABTS•^+^ solution and Te, Ba, Dex/BTGd or ascorbic acid dispersions (100 µg/ml) were mixed. After incubation at 37 °C in the dark for 5 min, the absorbance of the mixtures was recorded at 734 nm. The ABTS•^+^ scavenging activity (%) was calculated using the following equation:

(2) $$\text{ABTS}{•}^{+}\;scavenging\;rate\;\left(\%\right)=\left[1-\frac{B_{1}-B_{2}}{B_{0}}\right]\times \;100\%$$

where *B_1_, B_2_* and *B_0_* are the absorbance of the sample, the sample blank, and the control, respectively.

#### 2.7.4 Nitric oxide radical (NO•) scavenging assay

Griess Reagent A (10 mg/ml sulfanilic acid) and Griess Reagent B (1 mg/mL *N*-1-naphthylethylenediamine hydrochloride) were freshly prepared. Subsequently, 200 µl NaNO_2_ (100 µM) was mixed with 200 µl Te, Ba, Dex/BTGd or ascorbic acid dispersions (100 µg/ml) and incubated at room temperature for 1 h. Thereafter, 100 µl Griess Reagent A and 100 µl Griess Reagent B were added sequentially, and the mixtures were allowed to react for 10 min at room temperature. The absorbance was then recorded at 540 nm using a UV–Vis spectrophotometer. The NO• scavenging activity (%) was calculated using the following equation:

(3) $$NO•\;scavenging\;rate\;\left(\%\right)=\left[1-\frac{C_{1}-C_{2}}{C_{0}}\right]\times \;100\%$$

where *C_1_, C_2_* and *C_0_* are the absorbance of the sample, the sample blank, and the control, respectively.

### 2.8 Antibacterial experiment

*Escherichia coli* (*E. coli*), *Staphylococcus aureus* (*S. aureus*) and *Pseudomonas aeruginosa* (*P. aeruginosa*) were purchased from Guangdong Microbial Culture Collection Center (GDMCC) and cultured in LB broth. Bacterial working solutions were prepared by diluting bacteria in the logarithmic growth phase 1:1000 in fresh LB broth.

#### 2.8.1 Construction of Te-resistant bacterial strains

A 10 µl aliquot of the bacterial working suspension was uniformly spread onto Te‑containing solid medium (20 µg/ml) and incubated at 37 °C for 24 h. Well‑isolated single colonies were subsequently inoculated into fresh LB broth for enrichment. After 24 h of incubation, an equal volume of fresh medium containing 40 µg/mL Te was added to the culture to obtain a final Te concentration of 20 µg/mL, followed by a further 24 h of incubation. This medium‑replacement procedure was then repeated with progressively increasing Te concentrations (40, 50, 60, 70 and 80 µg/ml).

#### 2.8.2 Antibacterial activity evaluation

Equal volumes of bacterial suspensions of *E. coli, S. aureus* and *P. aeruginosa* were mixed with serial concentrations of Te, Ba or Dex/BTGd to obtain final drug concentrations of 250, 125, 62.5, 31.25 and 15.625 µg/ml. The mixtures were dispensed into 96‑well plates and incubated at 37 °C for 24 h. Optical density at 600 nm was recorded at 0 and 24 h using a microplate reader (TECAN Infinite F200). The bacterial survival rate was calculated according to the following formula:

(4) $$Survival\;rate\;\left(\%\right)=\frac{A_{1}-A_{0}}{B_{1}-B_{0}}\times \;100\%$$

where *A_0_* and *A_1_* represent the absorbance values of the experimental group at 0 h and 24 h, respectively, and *B_0_* and *B_1_* represent the absorbance values of the control group at 0 h and 24 h, respectively.

The dose-response curves were fitted using GraphPad Prism software 10.1.2, with the logarithm of drug concentration (log_10_C) as the abscissa and the corresponding survival rate as the ordinate. The half-maximal inhibitory concentration (IC_50_) of each drug, defined as the drug concentration required to inhibit 50% of bacterial growth, was calculated via the regression equation. The combination index (CI) rate was calculated using the following formula:

(5) $$CI=\frac{IC_{50\;\left(\text{Te}\;\text{in}\;\text{combination}\right)}}{IC_{50\;\left(\text{Te}\;\text{alone}\right)}}+\frac{IC_{50\;\left(\text{Ba}\;\text{in}\;\text{combination}\right)}}{IC_{50\;\left(\text{Ba}\;\text{alone}\right)}}$$

A CI < 1 indicates synergistic effect, CI = 1 indicates additive effect, and CI > 1 indicates antagonistic effect.

#### 2.8.3 Bacterial plate count assay

Bacterial suspensions were mixed 1:1 (v/v) with Te, Ba or Dex/BTGd dispersions. Additionally, corresponding concentrations of Gd^3+^, mixture of Ba and Gd^3+^ (Ba + Gd^3+^), Dex, and mixture of Dex, Ba, Te and Gd^3+^ (Dex + Ba + Te + Gd^3+^) were also examined. The mixtures were incubated at 37 °C with shaking at 150 rpm for 12 h. Subsequently, the cultures were diluted 1000‑fold in fresh LB broth, and 10 µl diluted suspension was evenly spread onto solid LB agar plates. The plates were incubated at 37 °C for 24 h. The bacterial growth inhibition rate was calculated as follows:

(6) $$Bacterial\;inhibition\;rate\;\left(\%\right)=\frac{C_{0}-C_{1}}{C_{0}}\times \;100\%$$

where *C_0_* and *C_1_* represent the number of colonies in the control and experimental groups, respectively.

#### 2.8.4 Biofilm inhibition assay

The working suspensions of *E. coli, S. aureus* or *P. aeruginosa* were mixed with an equal volume of fresh LB broth containing 100 µg/ml Te, Ba or Dex/BTGd and dispensed into 96‑well microtiter plates. Following incubation at 37 °C for 24 h under static conditions to allow biofilm formation, the culture medium was carefully aspirated and the wells were gently rinsed with PBS to remove nonadherent cells.

Subsequently, 100 µl methanol was added to each well to fix the biofilms, and the plates were incubated for 15 min. After removal of methanol and air‑drying, 1% (w/v) crystal violet solution was added and the plates were incubated for 5 min at room temperature. The excess dye was then removed, the wells were gently washed with PBS, and 100 µl of 33% (v/v) glacial acetic acid was added to each well to solubilize the bound crystal violet, followed by incubation at 37 °C for 30 min. The absorbance was recorded at 590 nm using a microplate reader, and the biofilm inhibition rate was calculated as follows:

(7) $$Biofilm\;inhibition\;rate\;\left(\%\right)=\left[1-\frac{D_{0}-D_{1}}{D_{0}}\right]\times \;100\%$$

where *D_0_* and *D_1_* represent the absorbance values of the control and experimental groups, respectively.

Additionally, the inhibitory effects of the different treatment groups on bacterial biofilms were examined using a confocal laser scanning microscope (CLSM, Leica TCS SP8, Germany). Bacterial biofilms were stained with a LIVE/DEAD bacterial staining kit, and imaging was performed at the same concentrations used in the treatments.

### 2.9 Cellular experiments

#### 2.9.1 Cytotoxicity assay

NIH/3T3 or RAW 264.7 cells in the logarithmic growth phase were trypsinized and seeded into 96‑well plates. After incubation for 12 h, the cells were exposed to various concentrations of Te, Ba or Dex/BTGd (100, 50 and 25 µg/ml) for 24 h. Cell viability was then assessed using the MTT assay [22]. In a separate experiment, NIH/3T3 cells seeded in 96‑well plates were treated with PBS, Te, Ba or Dex/BTGd (25, 50 or 100 µg/mL) in the presence of H_2_O_2_ (400 µM). A control group without H_2_O_2_ was also included. After 24 h of treatment, cell morphology was observed under an inverted microscope, and cell viability was determined using the MTT assay.

#### 2.9.2 Cell migration assay

NIH/3T3 cells were inoculated into 6-well plates and cultured until the desired level of fusion was achieved. A straight scratch was made in the cell monolayer using a 1000 µl sterile pipette tip. After washing with PBS to remove detached cells, the original medium was replaced with fresh medium containing 50 µg/ml of Te, Ba or Dex/BTGd. Images of the scratch were captured at 0 h and 24 h. The migration rate was quantified using ImageJ software and calculated as follows:

(8) $$Migration\;rate\;\left(\%\right)=\frac{M_{1}-M_{0}}{M_{0}}\times \;100\%$$

where *M_0_* is the scratch area at 0 h, and *M_1_* is the scratch area at 24 h.

#### 2.9.3 Measurement of intracellular total ROS and MDA concentration

NIH/3T3 cells were seeded into 12-well plates, then assigned to the following treatment groups: PBS, Te, Ba and Dex/BTGd, each in combination with 400 µM H_2_O_2_. The final concentrations of Te, Ba and Dex/BTGd were 50 µg/ml. After 12 h of treatment, cells were stained with DCFH-DA (5 µM), and the intracellular ROS levels were detected by flow cytometry.

The same experimental groups as described above were applied. After 12 h of treatment, cells were collected and the MDA content was measured using a commercial MDA assay kit according to the manufacturer's instructions.

#### 2.9.4 Catalase (CAT)-like activity assay

NIH/3T3 cells were seeded onto coverslips in 12-well plates. Liquid paraffin was overlaid on the medium to create an oxygen-deprived environment. The culture medium was then replaced with fresh medium containing the following treatments: Control, Te, Ba and Dex/BTGd, each with or without H_2_O_2_. Cells were incubated with [Ru(dpp)_3_]Cl_2_ (5 µM) for 4 h under continuous liquid paraffin overlay. Subsequently, the cells were stained with Hoechst 33342 (10 µM), washed with PBS, and fixed with 4% paraformaldehyde. The cells were observed using a CLSM.

#### 2.9.5 Mitochondrial function assay

**Mitochondrial membrane potential (MMP) assay:** The same experimental groups as described in Section 2.9.3 were applied. After 24 h of incubation, the cells were stained with 5 µM Rhodamine 123, and the changes in MMP were analyzed by flow cytometry.

**Intracellular ATP assay:** Cells were treated as described above. After treatment, the cells were washed with ice-cold PBS and lysed. ATP levels were measured using an ATP Assay Kit according to the manufacturer's instructions.

#### 2.9.6 Cell apoptosis assay

The same experimental groups as described in Section 2.9.3 were applied. Following 24 h of treatment, cells were stained with Annexin V-FITC and PI for 30 min. Cell apoptosis was analyzed by flow cytometry.

#### 2.9.7 Macrophage polarization analysis

**Macrophage polarization (M1/M2) induction:** RAW 264.7 cells were seeded in 12-well plates. For M1 polarization, cells were stimulated with LPS (100 ng/ml) and IFN-γ (20 ng/ml); for M2 polarization, IL-4 (20 ng/ml) was added. After 24 h of incubation, cells were harvested and stained with CD80-APC and CD206-PE. Macrophage polarization was analyzed by flow cytometry.

**Macrophage repolarization assay:** M1-type macrophages induced as described above were incubated with fresh medium containing Te, Ba or Dex/BTGd at a concentration of 50 µg/ml for 24 h. The cells were then collected and subjected to the same staining and analytical procedures as above.

#### 2.9.8 Hemolysis test

Fresh blood was collected from 6-week-old female ICR mice and centrifuged to remove serum. A 2% (v/v) erythrocyte suspension was prepared using physiological saline. Equal volumes of the erythrocyte suspension were mixed with PBS (negative control), ddH_2_O (positive control), Te, Ba and Dex/BTGd at final concentrations of 50 µg/ml. The mixtures were incubated at 37 °C for 3 h, followed by centrifugation at 10,000 rpm for 3 min. The absorbance of the supernatant at 540 nm was measured using a microplate reader.

### 2.10 Animal experiments

#### 2.10.1 Animals

Female ICR mice (6–8 weeks) were purchased from the Wushi Experimental Animals Center (Fuzhou, China). All related animal experimental procedures were carried out according to the protocols approved by the Institutional Animal Care and Use Committee of Fuzhou University.

#### 2.10.2 Animal model establishment and treatment protocol

Mice were anesthetized by intraperitoneal injection of sodium pentobarbital (80 mg/kg). After gently retracting the tongue with sterile forceps to expose the trachea, 50 µl Te-resistant or wild-type *P. aeruginosa* suspension (5 × 10^6^ colony forming units) was intratracheally administered using a microsprayer aerosolizer.

Following bacterial inoculation, mouse behavior was monitored. At 6, 24 and 48 h post-infection, mice were administered saline, Te (8 mg/kg), Ba (8 mg/kg), Dex/BTGd (equivalent to 8 mg/kg Te and 8 mg/kg Ba) or dexamethasone (5 mg/kg) [23] combined with Te (8 mg/kg). The experimental groups were as follows: Healthy group, Saline* group, Te group, Te* group, Ba* group, Dex/BTGd* and Te + dexamethasone* group (where "*'' denotes treatment in Te-resistant strain-infected mice). All mice were euthanized at 72 h post-infection.

To evaluate the biodistribution of the nanoplatform, indocyanine green (ICG)-labeled Dex/BTGd was prepared by mixing ICG with the nanoplatform at an equal ratio. Free ICG was removed by dialysis against water. For *in vivo* fluorescence imaging, 100 µl ICG-labeled Dex/BTGd was intravenously injected into healthy mice or mice infected with Te-resistant *P. aeruginosa* (*n* = 3). Images were acquired at predetermined time points using a small animal fluorescence imaging system (PerkinElmer IVIS Lumina XR III, USA), and mean radiant efficiency was analyzed using IVIS Living Imaging 4.4 software.

#### 2.10.3 CT imaging and MRI

Thoracic CT scans were performed using a PHILIPS system (Netherlands) to compare pulmonary imaging differences among healthy mice, saline-treated controls, and mice infected with Te-resistant *P. aeruginosa*. During scanning, anesthesia was maintained with isoflurane, and vital signs were monitored. CT parameters were as follows: tube voltage = 80 kV, tube current = 50 mA, total scan time = 0.35 s.

For MRI experiments, Gd-DOTA and Dex/BTGd were used. A Siemens 3.0 T MRI system (SIEMENS, German) was employed for *T_1_*-weighted imaging of the thoracic cavity in mice infected with Te-resistant *P. aeruginosa* with the following parameters: TR = 650.0 ms, TE = 10 ms, Slice Thickness = 1.0 mm, Slice Spacing = 1.0 mm, FoV = 80 × 80 mm^2^. Imaging was performed both before and after contrast administration. Additional CT and MRI scans were performed on the lungs of mice injected with Dex/BTGd.

#### 2.10.4 Bacterial load in lung tissues

Lung tissues from each experimental group were aseptically excised. Homogenates were prepared in a biosafety cabinet using pre‑chilled sterile saline and autoclaved tissue grinders, followed by filtration through sterile cell strainers to remove debris. For quantification of the pulmonary bacterial burden, 20 µl of each homogenate was evenly spread onto LB agar plates and incubated at 37 °C for 24 h, after which colonies were counted to determine the bacterial load.

#### 2.10.5 Assessment of pulmonary edema

Fresh lungs from each treatment group were immediately excised and weighed to determine the wet weight. The lungs were then dehydrated through a graded ethanol series, followed by air-drying at room temperature, after which the dry weight was recorded. The dry-to-wet weight ratio (D/W ratio) was subsequently calculated as an index of pulmonary edema.

#### 2.10.6 Pathological analysis of lung tissues

Freshly collected lung tissues were immediately fixed in 4% paraformaldehyde for 24 h. Paraffin sections of 5 µm thickness were prepared and stained using H&E and Masson's trichrome kits according to the manufacturer's protocols. Slides were mounted with neutral balsam and imaged under light microscopy. At 72 h post-inoculation, the heart, liver, spleen, and kidneys were also collected and processed identically for H&E staining.

#### 2.10.7 Pulmonary macrophage subpopulation

Lung tissues from each treatment group were homogenized and filtered through cell strainers to obtain single-cell suspensions. Cells were stained with fluorescently labeled antibodies (FITC-CD11b, PE-CD80, APC-CD206) for 30 min, followed by flow cytometric analysis.

#### 2.10.8 TGF-β measurement

TGF-β levels were quantified using a commercial ELISA kit according to the manufacturer's instructions.

#### 2.10.9 Blood biochemistry analysis

Healthy mice were intravenously injected with normal saline and Dex/BTGd three times. Blood samples were collected via retro-orbital bleeding, and the concentrations of aspartate aminotransferase (AST), alanine aminotransferase (ALT), creatinine (CREA), and blood urea nitrogen (BUN) were quantified using commercial ELISA kits according to the manufacturer's instructions.

#### 2.10.10 Measurement of the residual Gd^3+^ concentration

At 72 h post-inoculation, the heart, liver, spleen, lung and kidney of mice were collected and homogenized. After centrifugation, the supernatant of each tissue homogenate was collected, acidified with 1% (v/v) nitric acid, and the residual Gd^3+^ concentration was quantified by ICP–OES.

### 2.11 Statistical analysis

All experimental data were analyzed using GraphPad Prism software 10.1.2. Quantitative data are presented as mean ± standard deviation (Mean ± SD). For comparisons between two groups, Student's *t*-test was employed. Multiple group comparisons were performed using one-way analysis of variance (ANOVA). A *p*-value of < 0.05 was considered statistically significant. Significance levels are indicated as follows: *P < 0.05, **P < 0.01, ***P < 0.001, ****P < 0.0001; ns indicates not significant.

## 3 Results and discussion

### 3.1 Self-assembly of BTGd

The phenolic hydroxyl and carboxyl groups in the molecular structure of Ba promote coordination with metal ions, thereby driving the formation of nanostructures. Previous studies have demonstrated that Ba possesses intrinsic antibacterial activity, and its coordination with Mn^2+^ not only enables the assembly of stable nanostructures but also significantly enhances antibacterial efficacy [18]. Te, as a broad-spectrum antibiotic with good clinical safety, can be administered via multiple routes; however, the rapid emergence of drug resistance has become an increasingly serious problem. Moreover, Te contains metal-chelating sites and shows a strong propensity to coordinate with various metal ions to form metal complexes [20]. Thus, the construction of composite nanoparticles based on Ba–metal ion–Te may provide a promising strategy to overcome antibiotic resistance. Gd^3+^ is commonly used in MRI in the form of chelates with ligands such as diethylenetriaminepentaacetic acid (DTPA) and 1,4,7,10-tetraazacyclododecane-1,4,7,10-tetraacetic acid (DOTA) [24]. These complexes serve as MRI contrast agents with improved relaxivity, enhanced stability, better clearance profiles, and reduced toxicity [25]. However, potential interactions between Gd^3+^ and either Ba or Te have not been investigated. Leveraging the metal-binding properties of Ba and Te, we designed an integrated Ba–Gd–Te composite system that combines therapeutic action against Te-resistant bacterial pneumonia with MRI contrast functionality.

To explore this, the interactions between Gd^3+^ and the different components were systematically examined. Slight precipitation occurred only in the Ba–Gd binary system (indicated by a yellow triangle in Fig. S1A), indicating a specific and strong Ba–Gd^3+^ interaction that may affect composite stability. DLS analysis (Fig. S1B) showed that Ba formed relatively large particles in water, consistent with its low solubility and consequent aggregation. Te dispersions displayed a bimodal distribution below 1000 nm and an additional peak above 1000 nm, indicating spontaneous yet unstable self-assembly. In the Te–Gd system, only a bimodal distribution below 1000 nm was observed, with disappearance of the >1000 nm peak, suggesting that Gd^3+^ both stabilizes Te assemblies and promotes formation of more stable Te nanostructures. Although the Ba–Te binary system exhibited a single peak, the particle size remained large (>500 nm). In contrast, the Ba–Te–Gd (BTGd) ternary system showed no visible precipitation and yielded well-dispersed nanocomplexes with diameters below 300 nm. Particle size distributions and PDI values for all groups are listed in Table S1. Together, these observations demonstrate that Gd^3+^ functions as an effective bridge, enabling the construction of a stable ternary nanostructure with favorable dispersion. Standard calibration curves relating concentration to absorbance were also established for Ba and Te (Fig. S2).

**Fig. 1:**
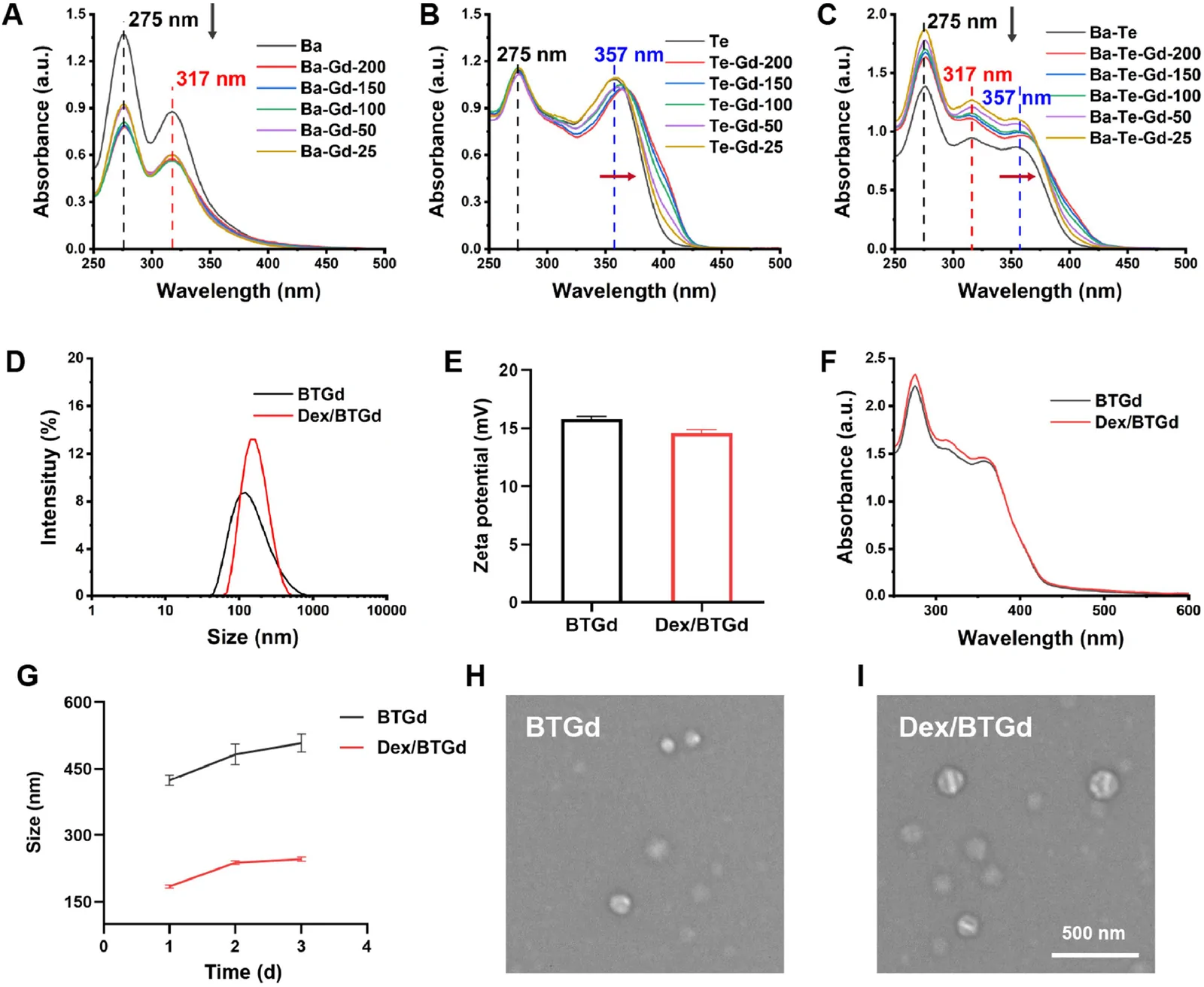
Synthesis and characterization of Dex/BTGd. (A-C) UV–Vis absorption spectra of Ba (A), Te (B) and the Ba-Te mixture (C) in the presence of increasing concentrations of Gd^3+^; (D) Hydrodynamic size distribution of BTGd and Dex/BTGd; (E) Zeta potential analysis of BTGd and Dex/BTGd; (F) UV–Vis absorption spectra of BTGd and Dex/BTGd; (G) Stability evaluation of Dex/BTGd and BTGd in PBS; (H, I) TEM images showing the morphology of BTGd (H) and Dex/BTGd (I).

The UV–Vis spectra were recorded while varying Gd^3+^ concentration in each system (Fig. 1A–1C). In the Ba–Gd mixture, the Ba absorption band at 275 nm progressively decreased with increasing Gd^3+^, whereas in the Te–Gd system, the 357 nm band underwent a redshift as Gd^3+^ concentration increased, indicating that both Ba and Te interact with Gd^3+^. In the ternary Ba–Te–Gd system, a simultaneous decrease at 275 nm and redshift at 357 nm were observed upon Gd^3+^ addition, demonstrating that the ternary complex preserves the interaction features of both Ba–Gd and Te–Gd binary systems and supports the formation of stable nanocomplexes.

To elucidate the assembly mechanism, intermolecular interactions were probed using competitive additives (Table S2). Addition of the anionic surfactant SDS or the nonionic surfactant Tween 20 markedly increased particle size, indicating that hydrophobic interactions are crucial for nanocomplex assembly. In contrast, the chelating agent EDTA disrupted the nanostructures by sequestering Gd^3+^ (Fig. S3), confirming that metal coordination is essential for structural integrity. Collectively, these findings indicate that BTGd self-assembly is primarily governed by hydrophobic interactions and Gd^3+^-mediated coordination, with Gd^3+^ acting as a structural bridge that binds Ba and Te, promoting the formation of stable nanostructures.

### 3.2 Preparation and characterization of Dex/BTGd

By surface modification, Dex-coated BTGd nanocomplexes (Dex/BTGd) were successfully prepared. Dex coating increased the hydrodynamic diameter from 158.63 ± 4.41 nm to 175.43 ± 4.10 nm (Fig. 1D) and slightly reduced the zeta potential from 15.80 ± 0.20 mV to 14.57 ± 0.32 mV (Fig. 1E), likely due to the slight negative charge of Dex in aqueous solution. UV–Vis spectra (Fig. 1F) showed no obvious changes after Dex coating, indicating that the BTGd core structure remained intact. The colloidal stability of Dex/BTGd and unmodified BTGd was compared in PBS over 72 h (Fig. 1G). BTGd showed poor stability, with evident aggregation within 24 h (size > 300 nm) and continuous growth thereafter. In contrast, Dex/BTGd maintained a size below 300 nm throughout the observation period, demonstrating markedly improved stability. This enhanced stability highlights the effectiveness of Dex coating and supports its suitability for biomedical applications. TEM images confirmed spherical morphologies for both BTGd and Dex/BTGd, with particle diameters consistent with DLS measurements (Fig. 1H and 1I).

To assess the broader applicability of this assembly strategy, the Ba component in Dex/BTGd was replaced with a series of natural products previously reported to have anti-inflammatory activity. These compounds are riched in hydroxyl or methoxy groups, which confer strong antioxidant properties and promote coordination with metal ions [26]. To further evaluate the versatility of tetracycline-based antibiotics in this system, Te was replaced with oxytetracycline or doxycycline, while Ba was substituted with mangiferin, quercetin, EGCG or tannic acid (Fig. S4). DLS analysis confirmed nanostructure formation in all formulations (Table S3). Although the component ratios were kept consistent across groups, the replacement of single functional components, which possess distinct physicochemical properties such as charge and hydrophobicity, led to variations in particle size due to their differential effects on the self-assembly process. Notably, the low standard deviations of particle size and PDI within each group confirm the excellent reproducibility of the synthesis protocol. These results suggest that further optimization of component ratios could improve particle size uniformity. UV–Vis absorption spectra were obtained for each group, and characteristic peaks were assigned (Fig. S5). Comparison of spectra for individual components and their corresponding nanocomplexes revealed shifts similar to those observed for Dex/BTGd (Fig. 1A), except in groups T3, T5 and O5, further supporting successful nanocomplex construction. The atypical spectral behavior of T3, T5 and O5 may arise from differences in binding sites between the substituted molecules and Gd^3+^ relative to the original Ba–Te system, although the exact mechanism remains to be clarified. The proposed assembly strategy exhibits substantial substrate tolerance and provides a promising platform for the rational design of nanodrug delivery systems.

### 3.3 Gd^3+^ release and in vitro MRI performance

**Fig. 2:**
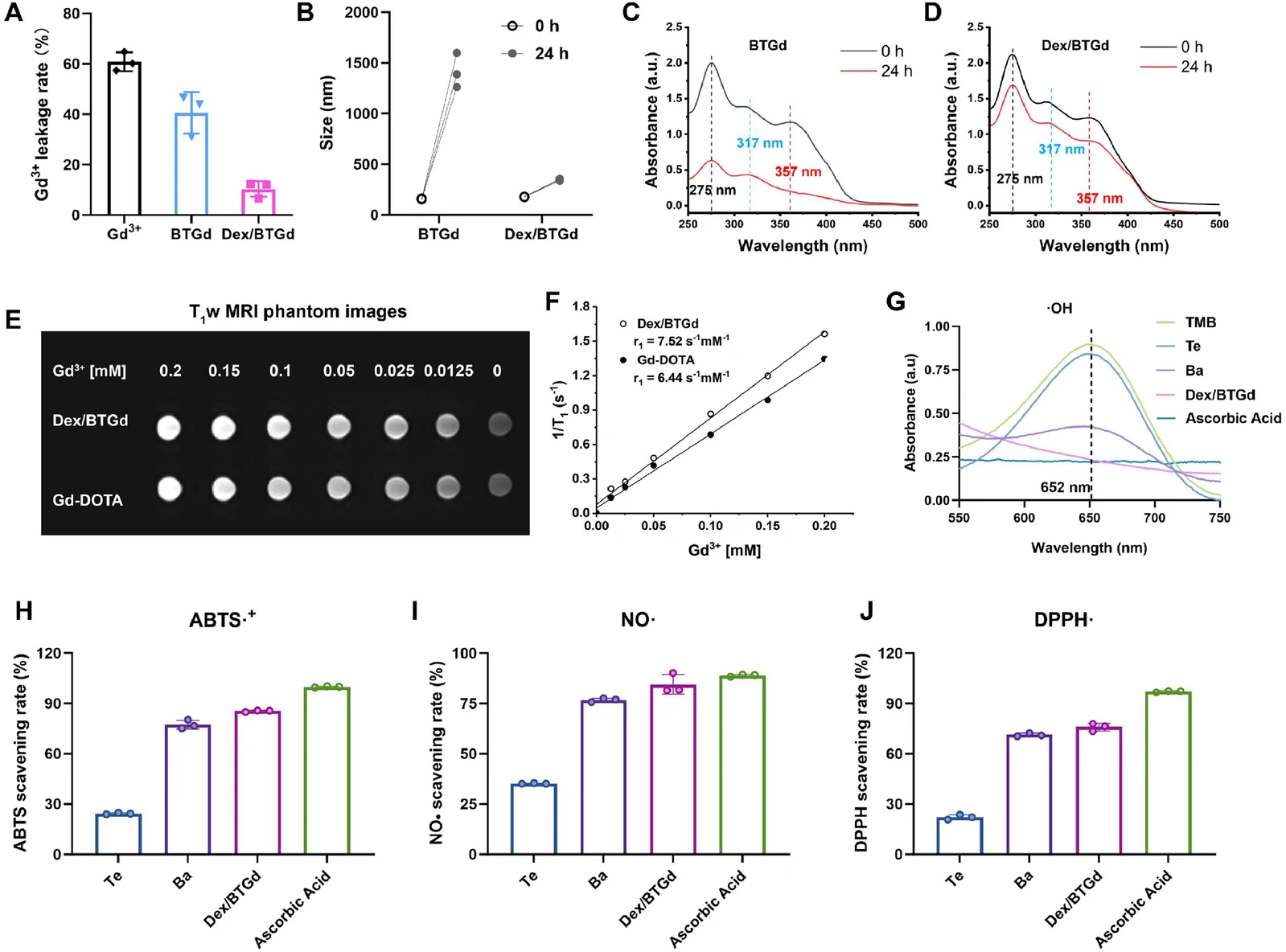
MRI performance and antioxidant properties of Dex/BTGd. (A) Cumulative release percentage of Gd^3+^ ions over 24 h in PBS; (B) Size change of BTGd and Dex/BTGd before and after Gd^3+^ release; (C, D) UV–Vis spectra change of BTGd and Dex/BTGd before and after Gd^3+^ release; (E-F) *T_1_*-weighted MRI phantom images acquired at 3.0 T and corresponding longitudinal relaxivity (*r_1_*) calculations; (G-J) Free radical scavenging assays evaluating antioxidant activity against hydroxyl radicals (•OH), ABTS radicals (ABTS•^+^), nitric oxide radicals (NO•) and DPPH radicals (DPPH•).

The clinical safety of Gd-based MRI contrast agents is constrained by the risk of free Gd^3+^ release, particularly in patients with renal impairment, which may trigger nephrogenic systemic fibrosis or gadolinium deposition [16]. We measured the release of Gd^3+^ from the nanocomplexes. A standard curve of Gd^3+^ concentration *versus* absorbance (0.1–10 µg/ml) was established, showing excellent linearity (Fig. S6). Accelerated dissociation studies were conducted at 37 °C with shaking at 120 rpm, and Gd^3+^ release was monitored for 24 h. The cumulative release rates were 60.78% ± 3.77% for free Gd^3+^, 40.49% ± 8.28% for BTGd, and 10.33% ± 3.20% for Dex/BTGd (Fig. 2A), indicating that nanocomplex encapsulation markedly suppressed Gd^3+^ leakage, with Dex surface modification providing additional protection. The particle size of BTGd increased from 157.50 nm to approximately 1400.00 nm after 24 h of shaking, whereas Dex/BTGd increased only from 177.10 nm to 344.30 nm (Fig. 2B). Dex modification not only inhibited aggregation but also significantly decreased the propensity for Gd^3+^ dissociation. UV–Vis spectra (Fig. 2C and 2D) further verified the superior stability of Dex/BTGd relative to BTGd. The Dex shell contributed to preserving structural integrity under mechanical stress and effectively sequestered Gd^3+^ ions. This dual stabilization effect substantially reduces the risk of Gd-related toxicity while maintaining therapeutic performance, supporting Dex/BTGd as a promising candidate for clinical translation as a safer MRI contrast agent.

MRI capability of Dex/BTGd was also evaluated. As shown in Fig. 2E, Dex/BTGd demonstrated comparable contrast enhancement to Gd-DOTA in MRI scans. Quantitative analysis (Fig. 2F) revealed a higher longitudinal relaxivity for Dex/BTGd (*r_1_* = 7.52 s^−1^·mM^−1^) than for Gd-DOTA (*r_1_* = 6.44 s^−1^·mM^−1^, consistent with reported values [27]). This improved performance is likely attributable to the combined effects of the large specific surface area of the nanoparticle, which enhances water access to Gd^3+^, and the hydrophilic Dex coating that promotes faster water exchange. We further investigated the effects of various ions (K^+^, Ca^2+^, Na^+^, Mg^2+^) on the *T_1_*-weighted MRI performance of Dex/BTGd. As shown in Fig. S7, the MRI capability of Dex/BTGd was not significantly affected under physiologically relevant ion concentrations, and the calculated relaxivity showed no obvious changes, indicating that Dex/BTGd could maintain stable *T_1_* imaging performance in complex physiological ionic environments and possess the potential for further *in vivo* imaging studies. The influence of pH on MRI performance was also examined. The results showed that the *T_1_* imaging capability of Dex/BTGd was significantly affected under acidic conditions, whereas no obvious changes were observed under physiological pH and alkaline conditions (Fig. S8). In addition, we found that the *T_2_* imaging performance of the nanoparticles was poor in all groups, regardless of Gd^3+^ concentration, which is consistent with the fact that Gd^3+^ primarily enhances *T_1_* relaxation. Therefore, *T_1_* imaging mode was selected for subsequent diagnostic experiments. Collectively, with its high physiological stability and greater longitudinal relaxivity than Gd‑DOTA, Dex/BTGd exhibits strong potential for clinical MRI diagnostics.

### 3.4 Antioxidant properties

Ba has been reported to exhibit antioxidant capacity [28]. On this basis, we hypothesized that Dex/BTGd would be capable of scavenging ROS. Several representative free radicals were selected to evaluate the radical-scavenging performance of Dex/BTGd, with ascorbic acid used as a positive control. A schematic of the underlying scavenging mechanisms is shown in Fig. S9. The hydroxyl radical (•OH) is a highly oxidizing species whose excess is harmful to biological systems. Ba group showed a marked decrease in absorbance at 652 nm (Fig. 2G), confirming efficient •OH scavenging. Notably, Dex/BTGd caused a further significant reduction in absorbance relative to Ba, indicating superior •OH elimination by the nanocomplex. The scavenging activities toward DPPH• and ABTS•^+^ were further examined and quantified from their characteristic absorption spectra. Both Ba and Dex/BTGd showed pronounced scavenging of multiple radicals, with Dex/BTGd exhibiting slightly higher efficiency than Ba (Fig. 2H–J). This enhancement is likely related to Gd^3+^ coordination, which may stabilize electron distribution and thereby increase antioxidant capacity [29]. In addition, Te displayed moderate radical-scavenging activity, weaker than that of the other groups, despite the presence of hydroxyl functional groups. Collectively, these findings provide additional experimental support for the potential use of Dex/BTGd in antioxidant-related applications.

### 3.5 Anti-bacterial activity

**Fig. 3:**
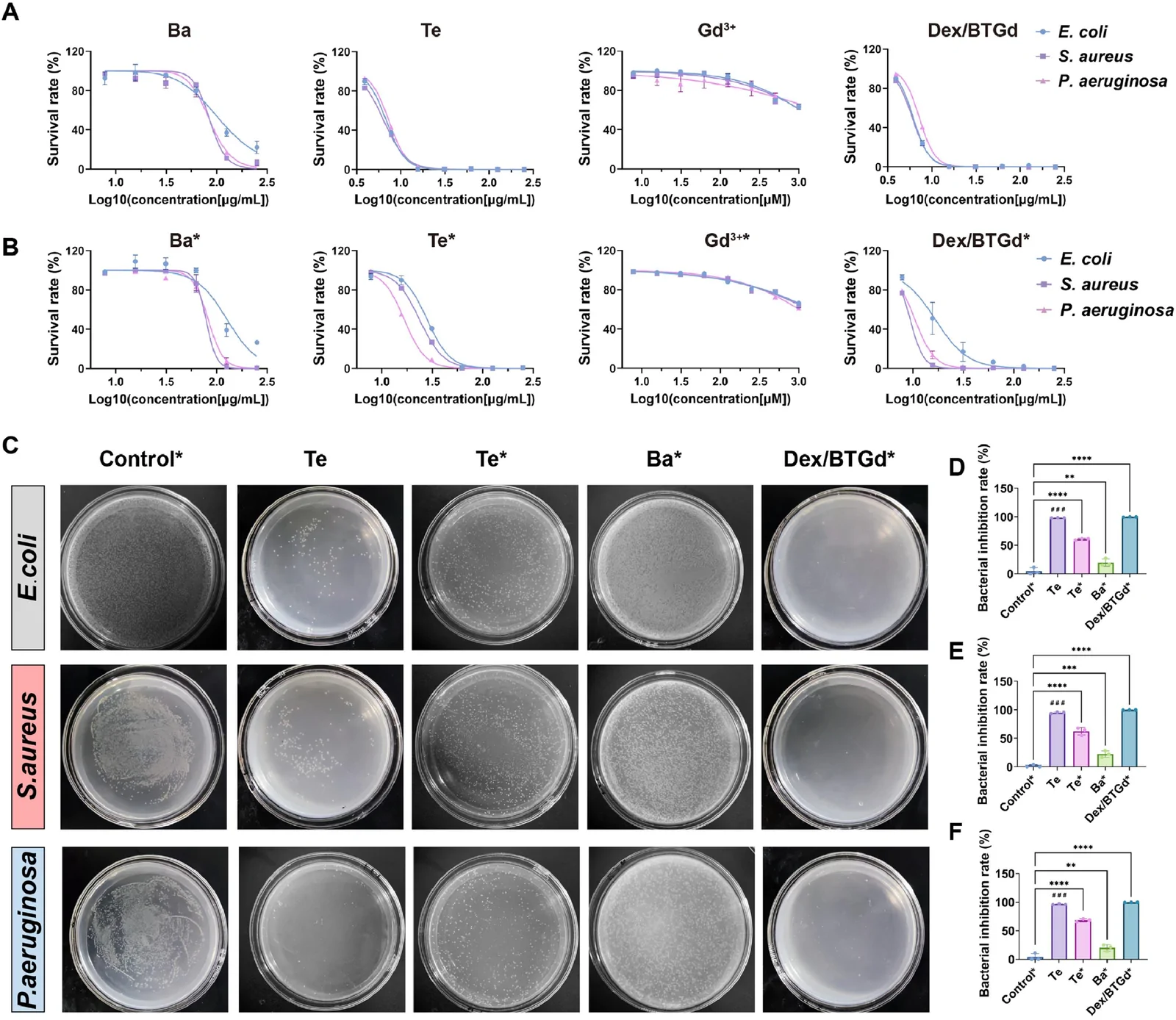
Evaluation of the antibacterial activity of Dex/BTGd. (A) Survival curves of wild-type *E. coli, S. aureus* and *P. aeruginosa*; (B) Survival curves of Te-resistant *E. coli, S. aureus* and *P. aeruginosa*; (C) Images of bacterial colonies; (D-F) Quantitative analysis of bacterial viability for (D) *E. coli*, (E) *S. aureus* and (F) *P. aeruginosa*. "*'' indicates drug-resistant strains. **P < 0.01, ***P < 0.001, ****P < 0.0001, compared with control. ^###^*P* < 0.001, compared with Te*.

Tetracycline antibiotics are broad-spectrum antimicrobial agents that act primarily by reversibly binding to the 30S ribosomal subunit of bacteria, thereby inhibiting bacterial protein synthesis [30]. Following the strategy illustrated in Fig. S10, we obtained Te-resistant strains of *E. coli, S. aureus* and *P. aeruginosa*. We then evaluated the effects of different concentrations of Te, Ba and Dex/BTGd on the survival of wild-type and resistant strains of these three species (Fig. 3A and 3B). Compared with the wild-type strains, the screened strains exhibited markedly higher survival under Te treatment, with *E. coli* showing the greatest resistance, followed by *S. aureus* and *P. aeruginosa*. Ba displayed minimal antibacterial activity against all three species at the tested concentrations, with significant inhibition only at 125 µg/ml, consistent with reports that Ba has poor low-dose antibacterial efficacy [31]. Notably, Dex/BTGd exhibited the strongest antibacterial activity among the tested treatments.

The minimum inhibitory concentration (MIC) values of Te, Ba and Dex/BTGd against both wild-type and resistant strains of *E. coli, S. aureus* and *P. aeruginosa* were subsequently determined (Table S4). Te exhibited low MIC values against the wild-type strains of all three bacteria. In contrast, Ba showed relatively high MIC values against all three strains, indicating poor antibacterial activity. Dex/BTGd demonstrated consistent MIC values of 15.625 µg/ml against all wild-type strains. After the development of resistance, the MIC values of Te increased significantly to 125 µg/ml, 62.5 µg/ml and 62.5 µg/ml for resistant *E. coli, S. aureus* and *P. aeruginosa*, respectively. Remarkably, Dex/BTGd maintained strong antibacterial activity against the resistant strains, with MIC values increasing only to 31.25 µg/ml for *E. coli* and *P. aeruginosa* while remaining unchanged for *S. aureus*. These results demonstrate the superior capability of Dex/BTGd to overcome Te resistance, likely attributable to the synergistic antibacterial effect between Ba and Gd^3+^. This observation is consistent with prior findings that the antibacterial activity of Ba can be significantly enhanced upon complexation with metal ions [31].

The plate coating method allowed intuitive observation of the inhibitory effects of each sample on bacterial growth (Fig. 3C). The results indicated that 50 µg/mL Te exhibited good antibacterial activity against all three wild-type strains, with the *P. aeruginosa* group showing the fewest colonies, suggesting higher sensitivity to Te. After treatment with Te, the colony counts of the three resistant strains did not decrease significantly, indicating markedly improved survival of the resistant strains in the presence of Te. In the Ba group, all resistant strains exhibited higher colony counts, confirming weak antibacterial efficacy at this concentration. In contrast, after treatment with Dex/BTGd, the three resistant strains formed very few colonies on the solid plates, demonstrating excellent antibacterial activity. Statistical analysis of colony counts and inhibition rates (Fig. 3D–3F) revealed that Te exhibited inhibition rates of 98.02%, 95.12% and 97.44% against wild-type *E. coli, S. aureus* and *P. aeruginosa*, respectively. In comparison, the inhibition rates against the resistant strains decreased to 60.62%, 62.46% and 68.17%, respectively. For the resistant strains treated with Ba, the inhibition rates were 19.72%, 22.52% and 20.06%, whereas the Dex/BTGd group showed inhibition rates exceeding 99%, further confirming its outstanding antibacterial capability.

To further clarify the mechanism underlying the enhanced antibacterial activity against drug-resistant strains, the IC_50_ values of Te, Ba and Dex/BTGd against both wild-type and drug-resistant strains of *E. coli, S. aureus* and *P. aeruginosa* were determined (Table S5). The CI values for Dex/BTGd against drug-resistant strains were all below 1, indicating a synergistic effect. The antibacterial effects of the individual components, their physical mixture, and Dex/BTGd against drug-resistant *P. aeruginosa* were compared, and the results are shown in Fig. S11. Compared with the control group, treatment with Te alone significantly inhibited the growth of drug-resistant *P. aeruginosa*, whereas Ba, Gd^3+^ or Dex alone exhibited no obvious inhibitory effect. However, the antibacterial activity was enhanced when Ba was combined with Gd^3+^. This enhancement can be explained by the coordination between Ba and Gd^3+^, which alters the overall charge distribution and hydrophobicity, facilitating electrostatic adsorption with negatively charged groups on the bacterial cell wall. This interaction increases membrane damage and permeability, promoting intracellular leakage and bacterial death. Notably, the antibacterial activity of the nanoformulation Dex/BTGd was significantly higher than the additive effect of the simple physical mixture (Dex + Ba + Te + Gd^3+^). This enhanced antibacterial activity is attributable to improved drug bioavailability, as nanoformulation increases the water solubility of the hydrophobic drugs Te and Ba, thereby promoting their uptake by bacteria [32].

Studies have shown that bacteria in biofilm states can exhibit tolerance to antimicrobial agents up to 100–1000 times greater than that of free-living bacteria [4]. Therefore, early intervention targeting biofilm formation during bacterial infections can improve bacterial clearance efficiency. Some studies have reported that Ba can inhibit the initial adhesion and aggregation of planktonic *S. aureus*, thereby suppressing biofilm formation [33]. However, in the aforementioned antibacterial experiments, Ba demonstrated relatively low antibacterial activity. Therefore, this study further evaluated its inhibitory effect on bacterial biofilm formation. The results showed that Te exhibited strong inhibitory activity against biofilms of wild-type *E. coli, S. aureus* and *P. aeruginosa* (Fig. S12). Quantitative analysis via crystal violet staining (Fig. S12A) revealed that the biofilm inhibition rate of Te against drug-resistant strains was significantly lower than that against wild-type strains, indicating its susceptibility to bacterial resistance. Although Ba demonstrated relatively low antibacterial activity, it moderately inhibited biofilm formation and performed slightly better than Te against resistant strains. Notably, Dex/BTGd was less affected by resistance and exhibited superior biofilm inhibition compared to both Te and Ba. Nevertheless, its biofilm inhibition rates against resistant strains remained lower than those of Te against wild-type strains. CLSM imaging (Fig. S12B) further validated these quantitative results. In the Te-treated group, fluorescence signals were attenuated in wild-type strains but remained strong in drug-resistant strains, reflecting its limited efficacy under resistant conditions. In contrast, treatment with Dex/BTGd resulted in obvious disruption of biofilm architecture and a significant reduction in fluorescence intensity across all three drug-resistant strains, directly confirming its potent inhibitory activity against biofilms formed by drug-resistant bacteria. In summary, despite the development of Te resistance in bacteria, the Dex/BTGd nanocomplex demonstrated promising biofilm-inhibitory effects, suggesting its potential application in the prevention and treatment of infections caused by drug-resistant bacteria.

### 3.6 Dex/BTGd alleviates oxidative stress damage and modulates cellular functions in vitro

#### 3.6.1 Exhibit excellent biocompatibility

**Fig. 4:**
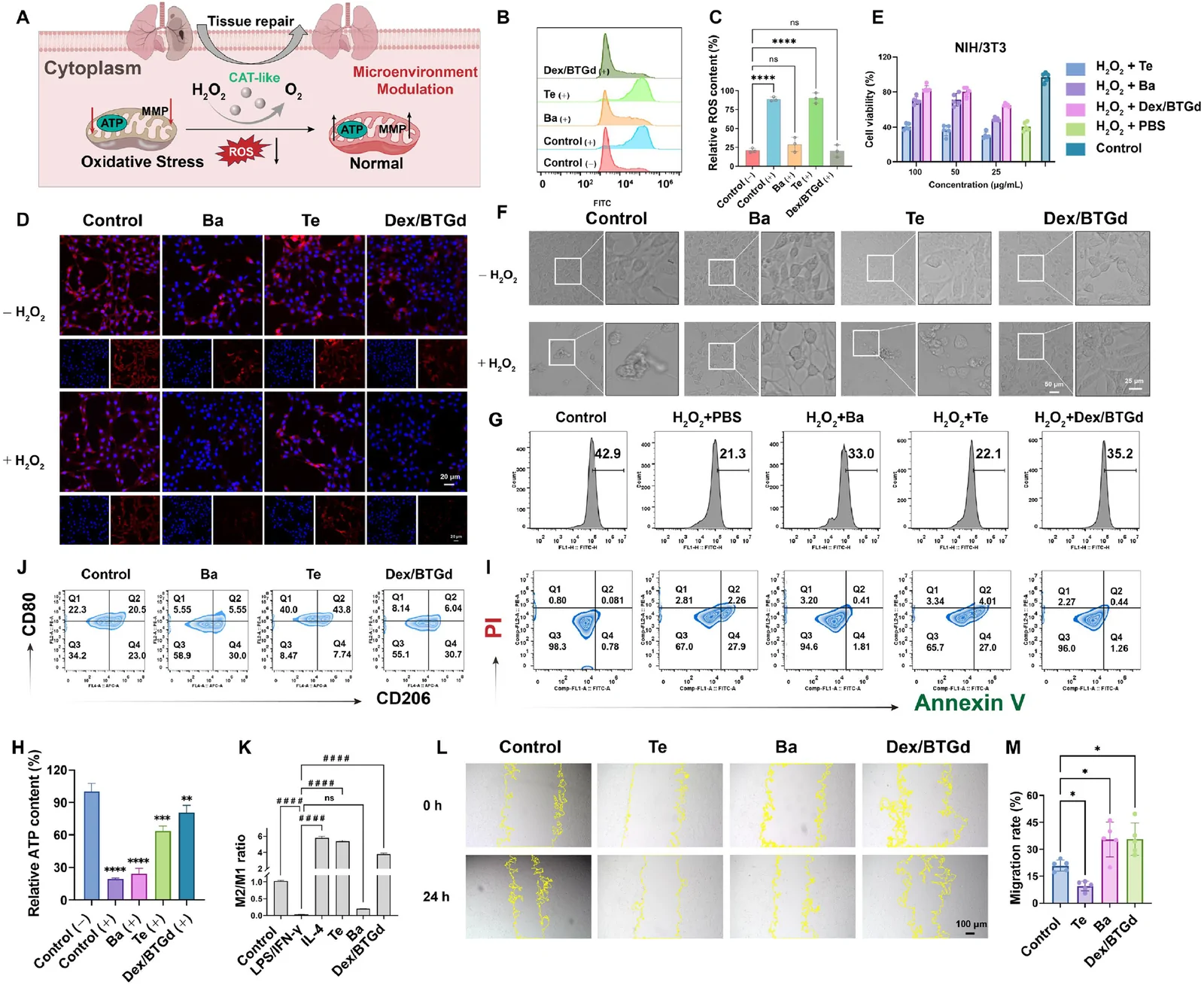
Cytoprotective effects and inflammation regulation. (A) Schematic illustration of the pulmonary inflammatory microenvironment and the cellular protection mechanism against injury; (B-C) Flow cytometric measurement of total ROS in NIH/3T3 cells after different treatments. + or – indicates the presence or absence of H_2_O_2_; (D) Intracellular oxygen levels detected by [Ru(dpp)_3_]Cl_2_ staining and visualized using confocal microscopy in NIH/3T3 cells after different treatments; (E-I) Assessment of cell viability (E), cell morphology (F), MMP (G), ATP production levels (H) and apoptosis (I) in NIH/3T3 cells under H_2_O_2_-induced oxidative stress with various treatments. + or – indicates the presence or absence of H_2_O_2_; (J-K) Macrophage polarization from M1 to M2 phenotype (J) and M2/M1 ratio (K) in RAW 264.7 cells across different treatment groups; (L-M) Images of migration (L) and quantitative analysis of migration rate (M) of NIH/3T3 cells under different treatments. **P < 0.01, ***P < 0.001, ****P < 0.0001, ns (not significant), compared with control. **^####^***P* < 0.0001.

During bacterial infection, excessive activation of immune cells can mediate inflammatory responses that result in irreversible histopathological damage. The local inflammatory microenvironment is typically marked by elevated ROS and the accumulation of inflammatory mediators. These conditions induce oxidative stress in tissue cells, promoting apoptosis and consequently increasing susceptibility to secondary bacterial infection. Thus, we examined the protective effects of Dex/BTGd on fibroblasts and its phenotypic regulation of macrophages under oxidative stress conditions (Fig. 4A). Given the critical role of fibroblasts in tissue repair under inflammation, we employed the widely recognized fibroblast cell line NIH/3T3. Additionally, the RAW264.7 macrophage cell line, which has been extensively utilized *in vitro* for studying phenotypic polarization and the balance between pro-inflammatory and anti-inflammatory responses [34], was employed to evaluate inflammatory modulatory effects.

The cytotoxicity of Dex/BTGd on normal cells was first evaluated using the MTT assay. The results showed that Te, Ba, Dex and Dex/BTGd at a concentration of 50 µg/ml all exhibited low cytotoxicity toward both NIH/3T3 and RAW 264.7 cells, with cell viability exceeding 80% (Fig. S13). This indicates that Dex/BTGd does not cause significant toxicity to normal cells while exerting its antibacterial effects, demonstrating favorable cellular compatibility. Additionally, Ba at a concentration of 25 µg/ml was found to promote cell proliferation, indicating its good biocompatibility.

To evaluate the *in vivo* application potential of the Dex/BTGd nano-system, its hemocompatibility was investigated. It should be noted that a hemolysis rate exceeding 5% significantly compromises biosafety. The results demonstrated that at the tested concentrations, Ba, Te and Dex/BTGd all exhibited hemolysis rates below 5% (Fig. S14), indicating favorable hemocompatibility and biosafety.

#### 3.6.2 Scavenge intracellular ROS and exhibit CAT-like activity

As described in antioxidant properties, both Ba and Dex/BTGd demonstrated excellent free radical scavenging capabilities. H_2_O_2_ is recognized as a harmful molecule that can impair cellular integrity and function. It serves as a crucial redox signaling molecule in oxidative stress and exhibits toxicity to various organisms [35]. We further examined the ability of Dex/BTGd to eliminate intracellular ROS. The results showed that treatment with Ba and Dex/BTGd significantly reduced total ROS levels in NIH/3T3 cells exposed to H_2_O_2_. Quantitative analysis revealed that the total ROS levels after Dex/BTGd treatment decreased markedly and showed no notable difference compared to the group without H_2_O_2_ addition (Fig. 4B and 4C). In contrast, the Te-treated group did not exhibit significant ROS-scavenging ability. In addition to ROS scavenging, we evaluated the effect of Dex/BTGd on lipid peroxidation by measuring intracellular MDA levels, a well-established marker of oxidative damage. As shown in Fig. S15, Dex/BTGd treatment significantly reduced MDA levels in H_2_O_2_-exposed cells, further supporting its effective intracellular antioxidant capacity. Collectively, these results demonstrate that Dex/BTGd effectively alleviates oxidative stress by both eliminating ROS and inhibiting lipid peroxidation.

Certain metal ions can catalyze Fenton or Fenton-like reactions with H_2_O_2_ to produce toxic •OH or exhibit CAT activity to decompose H_2_O_2_ into water and oxygen [36]. Given the effective ROS scavenging effects of Ba and Dex/BTGd, we hypothesized that they may display CAT-like activity under H_2_O_2_ conditions rather than generating •OH. To test this, we used the [Ru(dpp)_3_]Cl_2_ probe, which has an excitation wavelength of 455 nm and an emission wavelength near 613 nm. It undergoes fluorescence quenching in the presence of O_2_, allowing indirect detection of O_2_ generation via fluorescence quenching signals. To avoid interference from atmospheric O_2_, the liquid surface was sealed with liquid paraffin. As shown in Fig. 4D, the control group exhibited strong red fluorescence, confirming a hypoxic cellular environment. The red fluorescence intensity was significantly weaker in the Ba and Dex/BTGd groups compared to the Te group, indicating alleviated cellular hypoxia. Under H_2_O_2_ conditions, the red fluorescence nearly disappeared in the Ba and Dex/BTGd treatment groups, confirming their ability to catalytically decompose H_2_O_2_ into O_2_. In contrast, the Te-treated group maintained red fluorescence even in the presence of H_2_O_2_. These results directly demonstrate that the Dex/BTGd nanosystem can exhibit CAT-like activity in H_2_O_2_ environments, continuously decomposing H_2_O_2_ to supply O_2_ to cells, thereby effectively mitigating oxidative stress damage.

#### 3.6.3 Rescue cell viability under H_2_O_2_-induced oxidative stress

The site of bacterial infection is often characterized by intense inflammatory reactions accompanied by high levels of ROS. Certain bacteria actively release substantial amounts of H_2_O_2_ [8], further exacerbating ROS production and inducing oxidative stress in tissue cells. Elevated ROS levels are detrimental to the management of host infections. Thus, we first examined the effects of H_2_O_2_ on the viability of NIH/3T3 cells. As shown in Fig. 4E, under 400 µM H_2_O_2_ exposure, the viability of NIH/3T3 cells decreased to 40.53% of that in the control group. Compared to the H_2_O_2_-treated group, Ba treatment at a concentration of 50 µg/mL restored cell viability to 71.13%. Meanwhile, the Dex/BTGd treatment group exhibited a more pronounced protective effect, with higher cell viability recovery rates compared to the Ba group, reaching 80.17% at the same concentration. Notably, both Ba and Dex/BTGd groups showed a concentration-dependent increase in cell viability. In contrast, the Te-treated group showed no significant difference compared to the H_2_O_2_ group.

To further evaluate oxidative damage to cellular structures and the restorative effects of different treatments, cell morphology was examined (Fig. 4F). H_2_O_2_ treatment induced typical morphological features of oxidative injury, including reduced cell density, cell rounding, and vacuolation. The addition of Ba ameliorated these morphological abnormalities in NIH/3T3 cells, while the Dex/BTGd-treated group exhibited a more pronounced cytoprotective effect, with cell density and morphology closely resembling those of normal cells. In contrast, the Te group showed no such protective effect. These comparative results highlight the unique advantage of the Dex/BTGd nano-system in maintaining cellular structural integrity under oxidative stress.

#### 3.6.4 Preserve mitochondrial function and suppress apoptosis under oxidative stress

Mitochondria serve not only as cellular ``powerhouses'' but also as critical signaling hubs that regulate cellular fate. Maintaining their functional integrity under oxidative stress is essential for cellular homeostasis. Studies have shown that oxidative stress-induced mitochondrial dysfunction is closely associated with the pathogenesis of various major diseases, such as diabetes, Alzheimer's disease and cardiovascular disorders [37]. Therefore, we examined two key indicators of mitochondrial function: MMP and ATP production. As shown in Fig. 4G, H_2_O_2_ treatment led to a 21.6% decrease in MMP compared to the control group, indicating significant mitochondrial dysfunction. The H_2_O_2_ + Te group exhibited a 20.6% reduction in MMP, which was comparable to the H_2_O_2_ + PBS group. In contrast, the H_2_O_2_ + Ba group exhibited an 11.7% recovery in MMP relative to the H_2_O_2_ group. Notably, the H_2_O_2_ + Dex/BTGd group displayed only a 7.6% decline compared to the control, the smallest reduction among all treatment groups, highlighting its superior ability to preserve mitochondrial function under oxidative stress.

Approximately 90% of cellular ATP is generated by mitochondria via oxidative phosphorylation. As indicated in Fig. 4H, ATP levels in the H_2_O_2_ + PBS group dropped drastically to 19.31% of the control, while the H_2_O_2_ + Te group declined to 24.39%, confirming severe bioenergetic deficit. In contrast, the H_2_O_2_ + Ba and H_2_O_2_ + Dex/BTGd groups maintained ATP production at 63.45% and 80.57% of the control, respectively, both significantly higher than the H_2_O_2_ + PBS group. These findings align with the MMP results, collectively demonstrating the superior mitochondrial protective effects of the Dex/BTGd nanoplatform.

During bacterial infection, the H_2_O_2_ released by bacteria and the ROS produced during the inflammatory response can act synergistically to damage mitochondrial function. Since mitochondrial dysfunction is a key trigger of apoptosis, we further evaluated apoptosis levels in NIH/3T3 cells under H_2_O_2_ exposure after various treatments (Fig. 4I). The Q2 and Q3 quadrants represent late and early apoptotic cells, respectively. The results demonstrated that 400 µM H_2_O_2_ significantly induced apoptosis, with a total apoptosis rate of 30.16%. Treatment with Ba or Dex/BTGd markedly reduced the apoptosis rate, indicating strong anti-apoptotic effects. In contrast, Te failed to confer effective protection against apoptosis.

#### 3.6.5 Modulate macrophage polarization toward an anti-inflammatory phenotype

Macrophages exhibit dual regulatory roles in inflammation, where M1 macrophages promote inflammation via pro-inflammatory factors and ROS, whereas M2 macrophages facilitate resolution and tissue repair [38]. We employed LPS/IFN-γ and IL-4 as inducers to polarize macrophages toward M1 and M2 phenotypes, respectively. Experimental results confirmed that LPS/IFN-γ treatment significantly increased expression of the M1 marker CD80 (Q1 quadrant), whereas IL-4 treatment markedly induced expression of the M2 marker CD206 (Q4 quadrant) (Fig. S16), indicating the successful establishment of the polarization models.

During bacterial infections, macrophages tend to polarize toward the pro-inflammatory M1 phenotype to enhance phagocytic capacity and antibacterial activity for pathogen clearance. However, excessive M1 macrophage infiltration can trigger intense inflammatory responses, leading to tissue damage and exacerbation of infection-related pathological processes. To explore potential intervention strategies, we further evaluated the regulatory effects of Te, Ba and Dex/BTGd on the M1 macrophage phenotype. As shown in Fig. 4J-K, Ba treatment significantly downregulated CD80 expression while simultaneously increasing the proportion of the M2 marker CD206 [22], suggesting its ability to promote M1 to M2 polarization. In contrast, Te treatment did not significantly induce M2 polarization. The Dex/BTGd treatment group exhibited a phenotypic modulation trend similar to that of Ba, further supporting the efficacy of nano-platform in facilitating M1 to M2 conversion. This property holds significant therapeutic potential for modulating excessive inflammatory responses following bacterial infection and remodeling the inflammatory microenvironment.

#### 3.6.6 Promote fibroblast migration

Bacterial infections often impair local tissue repair, and compromised cell migration is a key factor hindering wound healing. Previous studies have reported that Ba can regulate the proliferation and migration of glial cells and Schwann cells [39]. Therefore, we further evaluated the effects of Te, Ba and Dex/BTGd on the migration ability of NIH/3T3 fibroblasts using a scratch assay. The wound closure rate was measured after 24 h. As shown in Fig. 4L and 4M, Ba significantly promoted NIH/3T3 cell migration, followed by Dex/BTGd. In contrast, Te showed a lower migration rate compared to the control, indicating its inhibitory effect on NIH/3T3 cell motility. According to the MTT assay data, Te exhibited low cytotoxicity toward NIH/3T3 cells at a concentration of 50 µg/ml, suggesting that the observed reduction in migration is mediated by a non-toxic mechanism. This finding is consistent with our previous report that Te could inhibit the migration of B16-F10 cells [21].

### 3.7 In vivo theranostic effects of Dex/BTGd in bacterial pneumonia

#### 3.7.1 Establishment and validation of murine model of P. aeruginosa pneumonia

ICR mice were subjected to tracheal instillation of either saline (control group) or *P. aeruginosa* bacterial suspension (infection group). Following bacterial inoculation, the mice exhibited decreased activity with diminished feeding and drinking. CT scans conducted at 12 h post-inoculation revealed distinct imaging patterns (Fig. S17): The healthy group showed clear pulmonary vascular contrast without abnormal shadows in the lung parenchyma; the saline control group demonstrated uniform mild hyperintensity in lung lobes, with signal intensity lower than that of vascular contrast; whereas the *P. aeruginosa*-infected group displayed extensively distributed high-density opacities throughout the lung lobes, indicative of inflammatory exudate accumulation in alveolar spaces. These radiographic characteristics are consistent with typical pathological changes of bacterial pneumonia, confirming the successful establishment of the murine pulmonary infection model.

#### 3.7.2 In vivo MRI performance

**Fig. 5:**
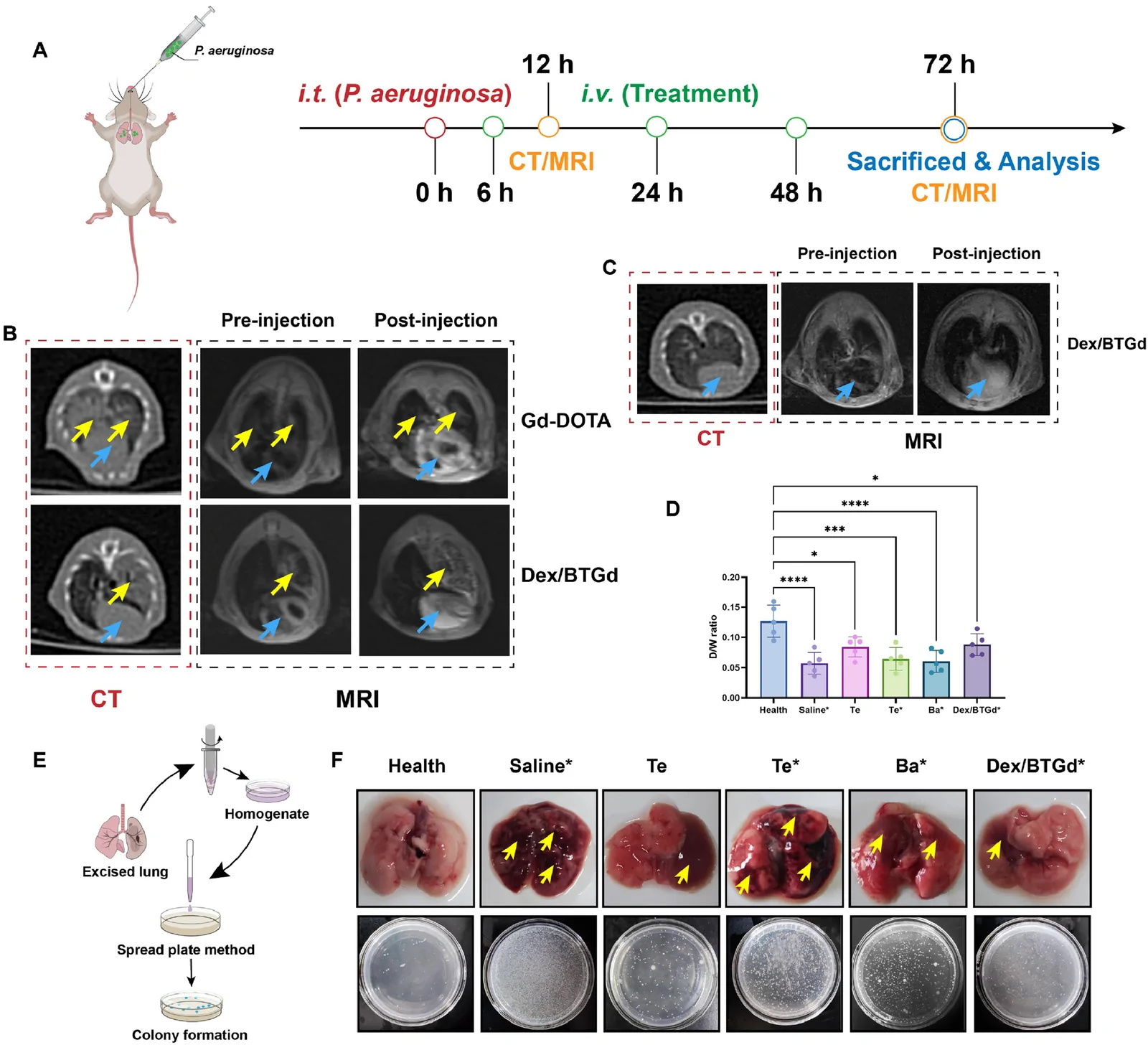
*In vivo* therapeutic efficacy. (A) Schematic illustration of the establishment of a mouse model of bacterial pneumonia and the overall experimental timeline; (B) CT scans of the lungs from pneumonia mice and corresponding transverse MRI images before and after injection of Gd-DOTA and Dex/BTGd (yellow arrows indicate lung lesions, blue arrows indicate heart. Images are presented with dorsal (top) and ventral (bottom) orientation for anatomical reference); (C) Lung CT and MRI images after three rounds of Dex/BTGd treatments; (D) Lung D/W weight ratios in each treatment group (* indicates drug-resistant strains; *n* = 5 mice per group); (E) Schematic of the bacterial load detection strategy in lung tissues; (F) Macroscopic lung appearance (yellow arrows indicate lung lesions) and bacterial culture results of lung samples from each treatment group at 72 h. *P < 0.05, ***P < 0.001, ****P < 0.0001.

Although CT imaging offers high resolution and rapid scanning, its reliance on X-ray radiation limits repeated use within short periods [15]. In contrast, MRI provides the advantages of no ionizing radiation and excellent soft tissue contrast, enabling higher image resolution and safer detection, though its imaging quality often relies on contrast agents [40]. We evaluated the MRI diagnosis capability of Dex/BTGd in mice. First, CT scanning confirmed the establishment of pulmonary infection (Fig. 5B). MRI examination without contrast agent injection showed low signal intensity in infected regions. Immediate MRI after injection of Gd-DOTA or Dex/BTGd revealed initial enhancement in the cardiac region, indicating that the contrast agents had entered the heart through blood circulation. The Gd-DOTA group showed minimal MRI signal enhancement in pulmonary infection areas, which diverged significantly from CT results, suggesting limited efficacy of this clinically contrast agent for bacterial infection localization. In comparison, the Dex/BTGd-injected group exhibited marked signal enhancement at infection sites, highly consistent with CT imaging, demonstrating its superior targeting capability. Consistently, we further evaluated the accumulation of nanoparticles in the lungs of normal *versus* infected mice via tail vein injection. The results showed significantly higher accumulation in the lungs of infected mice (Fig. S18), confirming the effective lung-targeting capability of Dex/BTGd. This difference may stem from the bacterial affinity provided by Dex modification on the Dex/BTGd surface, coupled with the enhanced permeability and retention (EPR) effect at inflammatory sites promoting nanoparticle accumulation, thereby significantly improving signal enhancement in infected regions. The results indicate that Dex/BTGd possesses excellent contrast-enhanced imaging capability for MRI diagnosis of bacterial pulmonary infections in mice. Fig. 5C displays pulmonary imaging following treatment with Dex/BTGd. CT imaging showed significant improvement in pathological lung conditions, with reduced parenchymal density and clearer contours. Subsequent MRI examination revealed no obvious lesion signals, suggesting alleviated pulmonary inflammation. These results demonstrate that Dex/BTGd exhibits favorable therapeutic efficacy against Te-resistant *P. aeruginosa* pulmonary infections in mice while maintaining excellent MRI diagnostic performance. To further validate the imaging accuracy, we performed H&E staining on the lung tissues of mice after MRI examination. Notably, the inflammatory lesion areas identified by H&E staining corresponded well with the lesion sites observed on *T_1_*-weighted MRI (Fig. S19), confirming the diagnostic accuracy of Dex/BTGd.

#### 3.7.3 In vivo antibacterial efficacy

After bacterial inoculation, different treatments were administered via tail vein injection at 6, 24 and 48 h. The selected doses (Te 8 mg/kg, Ba 8 mg/kg) were determined based on previously reported effective dose ranges for tetracycline derivatives in murine infection models (2–10 mg/kg) [41] and the "dose balance'' principle for multicomponent nanosystems to achieve synergistic antibacterial effects while minimizing systemic toxicity [42]. Lungs were harvested from each treatment group 72 h post-infection. To quantitatively assess pulmonary edema, we measured the D/W ratio of lung tissues, where higher values indicate less severe edema (Fig. 5D). The saline* group showed the most severe edema. Among drug-resistant infection groups, the Te* and Ba* groups exhibited higher edema levels than the Te and Dex/BTGd* groups. These results indicate that while Te shows good antibacterial efficacy against wild-type *P. aeruginosa* infection by effectively clearing bacteria and reducing tissue damage, its therapeutic effects are significantly limited against drug-resistant strains, failing to manage infection progression and tissue destruction. In comparison, Dex/BTGd demonstrated superior performance in the drug-resistant infection model, maintaining strong antibacterial activity while effectively improving pathological pulmonary edema. Fig. 5E illustrates the protocol for bacterial identification in lung tissues from each treatment group. The drug-resistant *P. aeruginosa* infection induced significant pulmonary inflammatory exudation and markedly elevated bacterial loads in lung homogenates (Fig. 5F). In the wild-type *P. aeruginosa* infection model, the Te treatment group showed reduced inflammatory exudation and significantly decreased bacterial loads. However, Te exhibited limited therapeutic efficacy against drug-resistant strains, while still displaying severe pulmonary damage and high bacterial burden. In contrast, the Ba* group demonstrated better pulmonary conditions than the Te* group, despite its weaker direct antibacterial activity, as evidenced by lower bacterial counts in lung homogenates. This phenomenon may be attributed to the ability of Ba to modulate the inflammatory microenvironment, thereby indirectly inhibiting bacterial invasion and promoting tissue repair. The Dex/BTGd* group exhibited synergistic effects, effectively clearing pathogens while significantly improving pulmonary pathology, demonstrating dual antibacterial and tissue-repair functions.

The anti-inflammatory and immunomodulatory effects of glucocorticoids have been proven to reduce the mortality of patients with severe community-acquired pneumonia [43]; however, they lack intrinsic antibacterial activity. To evaluate the clinical advantages of the Dex/BTGd nanoplatform constructed in this study, a Te and dexamethasone (Te + dexamethasone) combination therapy group was included as the control in the *in vivo* experiments. As depicted in Fig. S20, compared with the model control group, the Te + dexamethasone treatment only partially alleviated pulmonary congestion, with residual lung tissue injury still evident, and failed to effectively eradicate drug-resistant *P. aeruginosa*. In contrast, Dex/BTGd exhibited significantly superior therapeutic efficacy. While retaining potent antibacterial activity to eliminate drug-resistant pathogens, it synergistically ameliorated pulmonary pathological lesions, alleviated tissue edema, and facilitated tissue repair, achieving an overall curative effect better than the clinical dual therapy.

#### 3.7.4 Amelioration of bacterial infection-induced pulmonary fibrosis

**Fig. 6:**
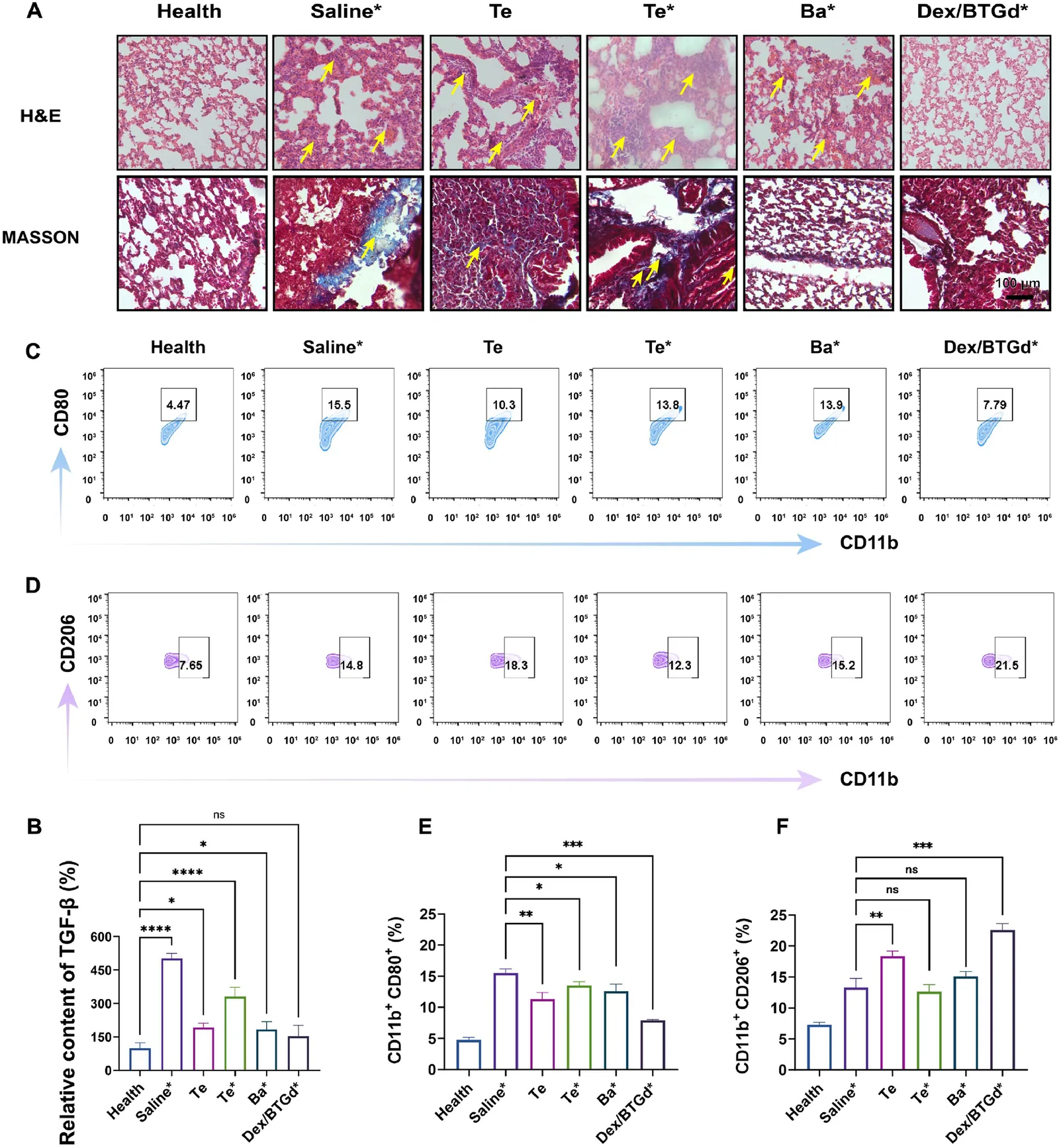
Dex/BTGd alleviates pulmonary fibrosis and modulates inflammatory responses. (A) Histopathological assessment of lung tissues from mice by H&E and Masson staining; (B) ELISA measurement of TGF-β levels in serum from mice in each treatment group; (C-D) Flow cytometry analysis of the M1 and M2 macrophages in the lungs from each treatment group; (E-F) Quantification of M1 and M2 macrophages in the lungs. *n* = 5 mice per group. *P < 0.05, **P < 0.01, ***P < 0.001, ****P < 0.0001; ns indicates not significant.

Bacterial pulmonary infections can cause severe tissue damage and induce pulmonary fibrosis [44]. To systematically evaluate the intervention effects of different treatments, histopathological staining of lung tissues was performed (Fig. 6A). H&E staining results showed that infection with drug-resistant *P. aeruginosa* induced typical features of acute lung injury, including significantly widened alveolar septa and localized areas of confluent consolidation. Masson staining further revealed substantial blue collagen deposition in the lung tissues, indicating notable fibrotic lesions. In wild-type *P. aeruginosa*-infected groups, H&E staining revealed localized pulmonary consolidation after Te treatment, while Masson staining detected diffuse fibrotic changes. Compared with wild-type infection group (Te group), the Te* group exhibited more severe pulmonary pathological damage and a significant increase in fibrosis. In contrast, the Ba* group showed persistent pathological alterations, such as thickened alveolar septa, and a partial reduction in fibrosis. Notably, the Dex/BTGd* group demonstrated the best tissue repair outcomes, with clear alveolar structure largely resembling normal lung morphology and the lowest degree of fibrosis. These results confirm that the Dex/BTGd regimen not only promotes effective tissue repair but also significantly suppresses fibrotic progression.

TGF-β plays a central role in the inflammation-fibrosis transition, and its dysregulation is a key driver of fibrotic diseases. The expression levels of TGF-β in the serum of mice from each treatment group were measured (Fig. 6B). The saline* group exhibited a significant increase in TGF-β levels, indicating a direct correlation between bacterial infection-induced severe inflammation and pronounced fibrotic lesions. Te treatment demonstrated weak antibacterial efficacy against Te-resistant *P. aeruginosa*, and the infection caused severe lung damage, resulting in persistently elevated TGF-β secretion, which likely contributed to the severe pulmonary fibrosis observed. In contrast, the Dex/BTGd* group markedly suppressed TGF-β expression. This finding is consistent with the reduced collagen deposition observed in Masson staining, confirming a dual mechanism where Dex/BTGd both inhibit pathogen growth and mitigate fibrosis via TGF-β regulation.

#### 3.7.5 Downregulation of pro-inflammatory macrophages in the lung

Macrophages play a dual role in inflammation, with M1 macrophages contributing to bacterial clearance [5], but their overactivation driving tissue damage and promoting bacterial dissemination. To further investigate the mechanism by which Dex/BTGd protects lung tissue and alleviates fibrosis, we analyzed macrophage subsets in the lungs of mice from different groups (Fig. 6C–6F). Treatment of wild-type infection with Te, which achieves effective bacterial clearance, led to a decreased proportion of M1 macrophages and an increased proportion of M2 macrophages. This shift indicates the initiation of lung tissue repair following successful pathogen eradication. In contrast, treatment of the drug-resistant strain with Te (Te* group) resulted in a higher proportion of M1 macrophages and a lower proportion of M2 macrophages compared to the wild-type infection group, suggesting a failure to resolve inflammation hindering tissue repair. Although *in vitro* cell experiments demonstrated that Ba effectively scavenged ROS and promoted M2 macrophage polarization, its poor *in vivo* antibacterial activity ultimately led to an increased M1 population due to uncontrolled infection. The Dex/BTGd* group exhibited optimal inflammatory modulation, with the lowest M1 and the highest M2 macrophage proportions. This outcome strongly correlates with its superior tissue-protective and anti-fibrotic effects.

#### 3.7.6 In vivo safety analysis

The *in vivo* safety of the treatments was assessed by histopathological analysis of major organs using H&E staining. As shown in Fig. S21, although the treatments produced varying therapeutic outcomes in infected lung tissues, none of the therapeutic regimens induced significant pathological changes in vital organs, including the heart, liver, spleen and kidneys, compared to the healthy control group. Notably, the Dex/BTGd treatment group demonstrated lung histopathology that was nearly indistinguishable from that of healthy controls. In addition, the residual Gd^3+^ content in the organs (heart, liver, spleen, lung and kidney) were measured by ICP–OES. The results showed that the Gd^3+^ levels in all tissues were below the detection limit, indicating minimal residual accumulation. Furthermore, blood biochemical indexes including AST, ALT, CREA and BUN showed no significant difference between the treatment group and the control group (Fig. S22), indicating excellent biosafety. These results confirm that the Dex/BTGd combination therapy offers excellent biosafety while ensuring high therapeutic efficacy.

### 3.8 Overall discussion and significance

Based on the inherent coordination properties between metal ions and phenolic hydroxyl/carboxyl groups, we successfully constructed a three-component self-assembled nanoplatform (Dex/BTGd) comprising Ba, Gd^3+^ and Te. To our knowledge, this is the first study utilizing Gd^3+^as a coordinating bridge to self-assemble a natural product and an antibiotic into stable nanoparticles. Beyond its potent antibacterial activity against drug-resistant strains, the nanoplatform efficiently scavenges excessive ROS, blocks oxidative stress-induced apoptosis, and promotes M2-biased macrophage polarization, thereby breaking the vicious cycle between infection and persistent inflammation. This triple synergistic mechanism, comprising pathogen clearance, microenvironment regulation, and tissue repair, distinguishes our work from conventional antibiotic monotherapy.

Dex/BTGd exhibits *in vivo* MRI performance comparable to Gd-DOTA alongside selective imaging of infection sites, a feature lacking in current clinical contrast agents. In a murine model of Te-resistant *P. aeruginosa* pneumonia, Dex/BTGd significantly reduced bacterial load, alleviated pulmonary edema and fibrosis, and suppressed TGF-β expression. The favorable hemocompatibility, low cytotoxicity, and minimal residual Gd^3+^ accumulation further support its biosafety. By integrating real-time diagnosis with multifaceted therapy into a single nanoplatform, this work provides a promising and clinically translatable strategy for managing drug-resistant bacterial pneumonia.

## 4 Conclusions

This work establishes a versatile nanoplatform, Dex/BTGd, which integrates diagnostic and therapeutic functions to address drug-resistant bacterial infections. Dex/BTGd was successfully constructed via the Dex-modified self-assembly of Ba, Te and Gd^3+^. Comprehensive *in vitro* and *in vivo* assessments confirmed the multifunctional efficacy of Dex/BTGd, including antibacterial, antioxidant, anti-inflammatory, and tissue-repair activities. This nanoplatform exerted potent inhibitory effects on both wild-type and Te-resistant pathogens by disrupting biofilms. Cellular experiments confirmed its significant antioxidant and anti-inflammatory effects, mitigating oxidative stress by scavenging free radicals and exhibiting CAT-like H_2_O_2_ decomposition activity, while also promoting the polarization of macrophages from the M1 to the M2 phenotype. In a murine model of Te-resistant *P. aeruginosa* pneumonia, Dex/BTGd significantly reduced the bacterial load, alleviated pulmonary edema and fibrosis, and enabled targeted MRI visualization of infected lesions. Mechanistically, Dex/BTGd operates by promoting anti-inflammatory macrophage polarization and downregulating TGF-β expression, thereby mitigating infection-associated inflammation and fibrosis. Compared with existing metal–organic framework (MOF)-based antimicrobial materials [45], Dex/BTGd integrates MRI-guided theranostics, targeted ROS scavenging, antibacterial activity, anti-inflammatory macrophage polarization, and tissue repair into a single system, offering a comprehensive strategy of diagnosis, treatment and repair for drug-resistant bacterial infections. Nevertheless, this study has several limitations. The scalability of the nanoplatform preparation remains to be optimized for large-scale production, and additional preclinical pharmacokinetic studies are necessary before clinical translation. Despite these constraints, our findings provide a proof-of-concept for the integration of imaging and synergistic anti-infective therapy, offering a valuable direction for future development.

## CRediT authorship contribution statement

**Xianbin Sun:** Writing – original draft, Data curation. **Xinyi Wang:** Investigation, Data curation. **Fangying Zheng:** Investigation. **Ruofei Xu:** Investigation. **Xinyi Shen:** Investigation. **Ding Tan:** Investigation. **Xudong Li:** Investigation. **Ya Wang:** Investigation. **Kaiming Peng:** Investigation. **Haijun Chen:** Supervision, Resources. **Xiumei Li:** Resources, Investigation. **Yu Gao:** Writing – review & editing, Supervision, Funding acquisition, Conceptualization.

## Conflicts of interest

The authors declare that there is no conflicts of interest.

## Appendix Supplementary materials

Image, application 1
