# Supplementary material for: A drug-repurposing nanotheranostic for synergistic management of resistant bacterial pneumonia
Source: Asian J Pharm Sci. 2026 Jul 11;21(4):101185. doi: 10.1016/j.ajps.2026.101185 (PMC13487832; doi:10.1016/j.ajps.2026.101185)
Supplement: Supplementary file 1 [file mmc1.docx]

**A Drug-Repurposing Nanotheranostic for Synergistic Management of Resistant Bacterial Pneumonia**

Xianbin Sun^1 #^, Xinyi Wang^1 #^, Fangying Zheng^1^, Ruofei Xu^1^, Xinyi Shen^1^, Ding Tan^1^, Xudong Li^1^, Ya Wang^1^, Kaiming Peng^3^, Haijun Chen^1,*^, Xiumei Li^2,*^, Yu Gao^1,*^

^1^Fujian Provincial Key Laboratory of Cancer Metastasis Chemoprevention and Chemotherapy, College of Chemistry, Fuzhou University, Fuzhou 350108, China.

^2^ Department of Radiology, The First Affiliated Hospital of Fujian Medical University, Fuzhou 350005, China

^3^ Department of Thoracic Surgery, Fujian Medical University Union Hospital, Fuzhou 350001, China

#X. Sun and X. Wang contributed equally to this work.

Corresponding authors:

*Yu Gao ( [hellogaoyu@126.com](mailto:hellogaoyu@126.com) or [ygao@fzu.edu.cn](mailto:ygao@fzu.edu.cn))

*Xiumei Li (meimei200110011@163.com)

*Haijun Chen ([chenhaij@gmail.com](mailto:chenhaij@gmail.com))

**Fig. S1**. (A) Photographic depictions of the solution states of different groups. (B) Particle size distribution analysis of different groups.

**Fig. S2**. UV–Vis absorption spectra and the corresponding calibration curves of Ba (A) and Te (B) showing the linear relationship between concentration and absorbance.

**Fig. S3.** Effects of different reagents on the particle size of BTGd. BTGd was introduced into aqueous solutions containing different concentrations of NaCl (100, 200, 500, 1000 mM), DMSO (5%, 10%, 15%, 20%, v/v), Urea (10, 20, 50, 100 mM), SDS (0.2%, 0.1%, 0.05%, 0.025%, w/v), Tween-20 (10, 20, 50, 100 mM), and EDTA (10, 20, 50, 100 mM). Each reagent was tested at four concentrations (Treat 1 to 4, with 1 being the lowest concentration and 4 the highest).

**Fig. S4**. Chemical structures of (**A**) the natural products and (**B**) the tetracycline-based antibiotics used in this study.

**Fig. S5.** UV–Vis characterization of natural compounds, antibiotics, and assembled nanocomplexes. (A) UV–Vis spectra and characteristic absorption peaks of the natural products. (B) UV–Vis spectra and characteristic absorption peaks of tetracycline-based antibiotics. (C) Spectral changes and peak shifts in UV–Vis absorption spectra after nanocomplex formation.

**Fig. S6**. Linear relationship between Gd^3+^ concentration and absorbance. The limit of detection (LOD) for Gd^3+^ was calculated as 0.0061747 μg/mL.

**Fig. S7**. Effect of various metal ions (K^+^, Ca^2+^, Na^+^, Mg^2+^) on the *T_1_*- and *T_2_*-weighted imaging performance and relaxivity of Dex/BTGd.

**Fig. S8**. Effect of different pH conditions on the *T_1_*- and *T_2_*-weighted imaging performance and relaxivity of Dex/BTGd.

**Fig. S9**. The mechanisms for detecting several typical free radicals.

**Fig. S10**. Strategy for inducing drug-resistant bacteria.

**Fig. S11**. (A) Images of bacterial colonies. (B) Quantitative analysis of bacterial viability against Te-resistant *P. aeruginosa*. **P* < 0.05,***P* < 0.01, ****P* < 0.001, *****P* < 0.0001.

**Fig. S12**. Biofilm inhibition assay ("*" indicates drug-resistant strains). (A) Quantification of biofilm formation by crystal violet staining. (B) CLSM images of bacterial biofilms stained with DMAO. **P* < 0.05, ** *P* < 0.01, *** *P* < 0.001; ns, not significant.

**Fig. S13**. Viability of NIH/3T3 and RAW 264.7 cells after treatment with different formulations.

**Fig. S14**. Hemocompatibility evaluation of different treatments in mouse erythrocytes.

**Fig. S15**. Intracellular MDA levels in NIH/3T3 cells after different treatments.

**Fig. S16**. Flow cytometry analysis of macrophage polarization induced by LPS/IFN-γ or IL-4.

**Fig. S17**. *In vivo* transverse CT imaging of lungs in mice from different treatment groups (yellow arrows indicate lung lesions; blue arrows indicate heart. Images are presented with dorsal (top) and ventral (bottom) orientation for anatomical reference.

**Fig. S18**. (A) Distribution of ICG-labeled nanoparticles in normal mice and mice with pulmonary bacterial infection at different time points after tail vein injection. (B) Corresponding quantitative fluorescence intensity curve of the lung.

**Fig. S19**. Transverse *T_1_*- and *T_2_*-weighted MRI images of mouse lungs before and after injection of Dex/BTGd, and the corresponding H&E-stained lung sections. Yellow arrows indicate lung lesions; blue arrows indicate heart. Images are presented with dorsal (top) and ventral (bottom) orientation for anatomical reference.

**Fig. S20**. Therapeutic effects of dexamethasone + Te combination and Dex/BTGd against Te-resistant *P. aeruginosa* pneumonia in mice. (A) Schematic illustration of the bacterial pneumonia mouse model and the overall experimental timeline. (B) Macroscopic lung appearance (yellow arrows indicate lung lesions). (C) Histopathological assessment of lung tissues from mice by H&E staining (yellow dashed boxes indicate lung lesions). (D) Bacterial culture results from lung samples.

**Fig. S21**. H&E staining images of major organs from mice in different treatment groups.

**Fig. S22**. Liver and kidney function in normal mice after three injections of saline and Dex/BTGd.

**Table S1**. Particle size and PDI values of different groups.

| **Group** | **Size (nm)** | **PDI** |
| --- | --- | --- |
| **Ba** | 1346.00 ± 50.03 | 0.16 ± 0.06 |
| **Te** | 340.60 ± 120.32 | 0.50 ± 0.01 |
| **Ba–Te** | 1001.77 ± 73.76 | 0.17 ± 0.07 |
| **Ba–Gd** | / | / |
| **Te–Gd** | 580.67 ± 395.82 | 0.68 ± 0.11 |
| **Ba–Te–Gd（BTGd）** | 158.63 ± 4.41 | 0.21 ± 0.02 |

**Table S2**. Reagents used to probe specific intermolecular interactions in the BTGd nanoparticle system.

| **Reagent** | Target Interaction |
| --- | --- |
| NaCl | Disrupts electrostatic interactions |
| DMSO | Disrupts π-π stacking |
| Urea | Disrupts hydrogen bonding |
| SDS | Disrupts hydrophobic interactions and hydrogen bonding |
| Tween-20 | Disrupts hydrophobic interactions |
| EDTA | Chelates metal ions (Gd^3+^) |

**Table S3**. Particle size and PDI values of various formulations.

| **Group** | **Size (nm)** | **PDI** |
| --- | --- | --- |
| T1 | 45.08 ± 5.73 | 0.26 ± 0.08 |
| T2 | 105.18 ± 6.31 | 0.22 ± 0.10 |
| T3 | 611.07 ± 7.99 | 0.23 ± 0.14 |
| T4 | 158.63 ± 4.41 | 0.21 ± 0.02 |
| T5 | 129.63 ± 1.45 | 0.14 ± 0.01 |
| O1 | 46.23 ± 1.64 | 0.38 ± 0.02 |
| O2 | 100.76 ± 2.48 | 0.12 ± 0.07 |
| O3 | 158.83 ± 1.46 | 0.11 ± 0.10 |
| O4 | 93.19 ± 2.19 | 0.26 ± 0.07 |
| O5 | 254.30 ± 3.50 | 0.26 ± 0.03 |
| D1 | 433.10 ± 69.97 | 0.27 ± 0.04 |
| D2 | 387.37 ± 26.70 | 0.37 ± 0.13 |
| D3 | 153.23 ± 25.16 | 0.16 ± 0.08 |
| D4 | 114.00 ± 16.64 | 0.34 ± 0.09 |
| D5 | 25.86 ± 2.75 | 0.26 ± 0.02 |

**Table S4**. MIC values of different treatment groups against tested bacteria ("*" indicates drug-resistant strains).

| **Bacteria** | **MIC (****μg/mL)** | | |
| --- | --- | --- | --- |
|  | **Te** | **Ba** | **Dex/BTGd** |
| ***E. coli*** | 15.625 | ＞250.000 | 15.625 |
| ***E. coli**** | 125.000 | ＞250.000 | 31.250 |
| ***S. aureus*** | 31.250 | 250.000 | 15.625 |
| ***S. aureus**** | 62.500 | 250.000 | 15.625 |
| ***P. aeruginosa*** | 31.250 | 250.000 | 15.625 |
| ***P. aeruginosa**** | 62.500 | 250.000 | 31.250 |

**Table S5**. IC_50_ values of different treatment groups against tested bacteria ("*" indicates drug-resistant strains).

| **Bacteria** | **IC_50_ (μg/mL)** | | | **CI** |
| --- | --- | --- | --- | --- |
|  | **Te** | **Ba** | **Dex/BTGd** |  |
| ***E. coli*** | 6.846 | 110.800 | 6.172 | 0.96 |
| ***E. coli**** | 28.060 | 126.500 | 16.640 | 0.72 |
| ***S. aureus*** | 6.413 | 86.150 | 6.008 | 1.01 |
| ***S. aureus**** | 23.580 | 78.170 | 9.392 | 0.52 |
| ***P. aeruginosa*** | 7.308 | 85.760 | 7.217 | 1.07 |
| ***P. aeruginosa**** | 16.660 | 82.280 | 10.460 | 0.75 |
